# The CD63 homologs, Tsp42Ee and Tsp42Eg, restrict endocytosis and promote neurotransmission through differential regulation of synaptic vesicle pools

**DOI:** 10.3389/fncel.2022.957232

**Published:** 2022-08-22

**Authors:** Emily L. Hendricks, Ireland R. Smith, Bruna Prates, Fatemeh Barmaleki, Faith L. W. Liebl

**Affiliations:** Department of Biological Sciences, Southern Illinois University Edwardsville, Edwardsville, IL, United States

**Keywords:** tetraspanins, CD63, endocytosis, glutamate, neuromuscular junction

## Abstract

The Tetraspanin (Tsp), CD63, is a transmembrane component of late endosomes and facilitates vesicular trafficking through endosomal pathways. Despite being widely expressed in the human brain and localized to late endosomes, CD63's role in regulating endo- and exocytic cycling at the synapse has not been investigated. Synaptic vesicle pools are highly dynamic and disruptions in the mobilization and replenishment of these vesicle pools have adverse neuronal effects. We find that the CD63 homologs, Tsp42Ee and Tsp42Eg, are expressed at the *Drosophila* neuromuscular junction to regulate synaptic vesicle pools through both shared and unique mechanisms. Tsp42Ee and Tsp42Eg negatively regulate endocytosis and positively regulate neurotransmitter release. Both *tsp* mutants show impaired locomotion, reduced miniature endplate junctional current frequencies, and increased endocytosis. Expression of human CD63 in *Drosophila* neurons leads to impaired endocytosis suggesting the role of Tsps in endocytosis is conserved. We further show that Tsps influence the synaptic cytoskeleton and membrane composition by regulating Futsch loop formation and synaptic levels of SCAR and PI(4,5)P_2_. Finally, Tsp42Ee and Tsp42Eg influence the synaptic localization of several vesicle-associated proteins including Synapsin, Synaptotagmin, and Cysteine String Protein. Together, our results present a novel function for Tsps in the regulation of vesicle pools and provide insight into the molecular mechanisms of Tsp-related synaptic dysfunction.

## Introduction

The Tetraspanin (Tsp) family of transmembrane proteins populate cell membranes and organize characteristic membrane landscapes known as Tsp-enriched microdomains (Stipp et al., 2003). At these sites, Tsps exert regulatory control over the spatiotemporal distribution of their binding partners (Charrin et al., 2014). Tsps mediate these interactions through well-conserved structural motifs, most notably the EC2 extracellular loop, which contains the characteristic CCG motif (Seigneuret et al., 2001; Kovalenko et al., 2005). Analyses of Tsp-binding proteins reveal interactions between Tsps and other transmembrane proteins, cell surface receptors, adhesion molecules, and intracellular signaling proteins (Hemler, 2005; Termini and Gillette, 2017). Therefore, Tsps act as orchestrators of cell signaling and extracellular interactions by maintaining structural organization of the cell membrane.

Tsps are expressed in diverse cell types and have wide-ranging physiological functions. For example, they contribute to immune function, reproduction, gastric cell regulation, and astrocyte differentiation (Kelić et al., 2001; Duffield et al., 2003; Stipp et al., 2003; Termini and Gillette, 2017; Zou et al., 2018; Jankovičová et al., 2020). Several Tsps are widely expressed in the central nervous system (Murru et al., 2018) but their neuronal functions are largely limited to descriptions of the organizational control that Tsps exert on cell membranes. For example, Tspan5, whose expression is enriched in the mammalian brain, facilitates dendritic spine maturation through regulation of transsynaptic cell adhesion molecule clustering (Moretto et al., 2019). Tspan7 interacts with protein interacting with C kinase 1 (PICK1) thereby regulating AMPA receptor trafficking (Bassani et al., 2012).

In addition to their role at the cell membrane, some Tsps function intracellularly. Specifically, the Tsp, CD63, is important for targeting KFERQ-containing peptides to exosomes in an ESCRT-independent manner (Ferreira et al., 2022). Furthermore, CD63 is highly enriched in exosomes (Escola et al., 1998) and late endosomal vesicles (Pols and Klumperman, 2009) and regulates the trafficking of synaptic proteins, including Synaptotagmin VII (Flannery et al., 2010) and the neurotrophin receptor p75 (Escudero et al., 2014) through endosomal pathways. Although vesicular trafficking is fundamental for basal cell function, it has extensive physiological implications at specialized sites like neuronal synapses with high rates of vesicle turnover (Saheki and De Camilli, 2012).

Disruptions in CD63 function are associated with a number of diseases and disorders impacting the brain like neuroblastoma progression (Chivet et al., 2014; Marimpietri et al., 2021), Herpes Simplex Virus 1 neuronal infection (Dogrammatzis et al., 2019), and neuronal dysfunction in Down syndrome (Gauthier et al., 2017). Additionally, CD63 is used as a platelet biomarker for advancing cognitive decline in Alzheimer's disease (Yu et al., 2021). Together, these clinical findings highlight the importance of CD63 in neurons. A better understanding of CD63's synaptic function would enhance our understanding of how Tsps are implicated in human health and disease.

To study the role of CD63 at the synapse, we used the glutamatergic *Drosophila* neuromuscular junction (NMJ), which, both structurally and functionally, resembles mammalian glutamatergic synapses (Chou et al., 2020). Human *CD63* gives rise to eight unique mRNA transcripts due to exon skipping and/or the use of an alternative start codon in exon 7. These alternative splice variants produce three human CD63 (hCD63) isoforms whose expression may confer tissue-specific functions (Hochheimer et al., 2019). In *Drosophila*, however, hCD63 orthologs are encoded by separate *tsp* genes that have diverse tissue expression profiles. Of the 37 *tsps* encoded by the *Drosophila* genome (Todres et al., 2000), we show that two CD63 orthologs, *tsp42Ee* and *tsp42Eg*, are expressed transsynaptically at the NMJ to functionally regulate locomotion and neurotransmitter release. We find that Tsps modulate these synaptic processes by influencing the localization of synaptic vesicle-associated proteins, including Synaptotagmin (Syt), Cysteine String Protein (CSP), and Synapsin (Syn), and by regulating cytoskeletal and membrane structure through the microtubule associated protein 1B (MAP1B)/Futsch, SCAR, and PI(4,5)P_2_. Our findings establish distinct roles for Tsp42Ee and Tsp42Eg in the maintenance of synaptic vesicle pools and negative regulation of endocytosis. Overall, these results uncover a novel role for the CD63 orthologs, *tsp42Ee* and *tsp42Eg*, in the regulation of neurotransmission and synaptic organization.

## Materials and methods

### Fly rearing and stocks

All fly stocks were reared at 25°C on Jazz Mix fly food (Fischer Scientific) in a Percival DR-36NL *Drosophila* incubator with an alternating 12 h light-dark cycle. Male and female third instar larvae or adult flies were used for all experiments. The following stocks were obtained from Bloomington *Drosophila* Stock Center: *w*^1118^ (RRID:BDSC_5905), *tsp42Ee*^*G*2619^ (RRID:BDSC_28119), *tsp42Ee*^*CC*01420^/*tsp42Ee-GFP* (RRID:BDSC_51558), *tsp42Eg*^*MB*08050^ (RRID:BDSC_25658), *UAS-hCD63* (RRID:BDSC_82215), *24B-Gal4* (RRID:BDSC_1767), and *elav*^*C*155^*-Gal4* (RRID:BDSC_458). The *tsp42Ee*^*G*2619^ and *tsp42Eg*^*MB*08050^ loss of function mutants were originally described in Bellen et al. (2011).

### Protein sequence accession and alignment

Tsp42Ee (NP_001260753.1), Tsp42Eg (NP_523633.1), and hCD63 (NP_001244318.1) reference protein sequences were obtained from NCBI and multiple sequence alignment was performed using EMBL-EBI Clustal Omega (v1.2.4) (Madeira et al., 2019). Aligned sequences were analyzed for residue similarity using the Sequence Manipulation Suite (written by Paul Stothard; bioinformatics.org/sms). References to similar amino acid residues use the following categorizations based on biochemical properties: ILV, FWY, KRH, DE, GAS, P, C, TNQM, with commas separating each group.

### Immunohistochemistry

Third instar larvae were filet dissected on 60 mm Sylgard-coated (World Precision Instruments) dishes in Roger's Ringer solution (pH = 7.15, 135 mM NaCl, 5 mM KCl, 4 mM MgCl${}_{2}^{*}$6H_2_O, 1.8 mM CaCl${}_{2}^{*}$2H_2_O, 5 mM TES, 72 mM sucrose, and 2 mM glutamate). Filet dissected larvae were fixed either in Bouin's fixative or 3.7% paraformaldehyde (PFA) for 30 min. Fixed larvae were placed in 1.5 mL centrifuge tubes containing PTX (PBS + 0.1% Triton X-100, Accuris Life Science Reagents; Integra Chemical) and washed in PTX for three 10-min intervals. Larvae were next washed in PBTX (PTX + 1% Bovine Serum Albumin, Fisher BioReagents) twice for 30 min. Primary antibodies included rabbit α-GFP (1:100, Torrey Pines Biolabs; RRID:AB_2313770), rabbit α-GluRIIC [1:5000, generated by Genscript using the sequence found in Marrus et al. (2004)], rabbit α-vGLUT [1:10,000, a gift from the Aaron DiAntonio lab (Daniels et al., 2004)], mouse α-Brp (1:50, Developmental Studies Hybridoma Bank; RRID:AB_2314866), mouse α-Syt1 (1:100, Developmental Studies Hybridoma Bank; RRID:AB_528483), mouse α-CSP (1:200, Developmental Studies Hybridoma Bank; RRID:AB_528183), mouse α-Syn (1:50, Developmental Studies Hybridoma Bank; RRID:AB_528479), mouse α-Futsch (1:100, Developmental Studies Hybridoma Bank; RRID:AB_528403), mouse α-SCAR (1:50, Developmental Studies Hybridoma Bank; RRID:AB_2618386), mouse α-WASp (1:10, Developmental Studies Hybridoma Bank; RRID:AB_2618392), rabbit α-Nwk (1:1000, a gift from the Kate O'Connor-Giles lab), and mouse α-PI(4,5)P_2_ (1:250, Echelon Biosciences, RRID:AB_427225). Primary antibodies were diluted in PBTX and incubated with larval tissues overnight at 4°C. Larvae then underwent three 10-min and two 30-min washes in PBTX. Secondary antibodies included α-mouse FITC (Jackson ImmunoResearch; RRID:AB_233558), α-rabbit FITC (Jackson ImmunoResearch; RRID:AB_2337972), and α-mouse TRITC (Jackson ImmunoResearch; RRID:AB_2340767) and were diluted 1:400 in PBTX and co-applied with Cy3- (RRID:AB_2338959) or A647-conjugated HRP (1:125, Jackson ImmunoResearch; RRID:AB_2338967 for 2 h at room temperature. Larvae were next washed with PBTX for three times for 10 min and two times for 30 min and placed on microscope slides and covered with Vectashield mounting medium (Vector Laboratories).

### FM 1-43FX labeling

FM 1-43FX labeling was performed as described (Verstreken et al., 2008). Briefly, third instar larvae were filet dissected in HL-3 without Ca^2+^ (pH = 7.2; 100 mM NaCl, 5 mM KCl, 10 mM NaHCO_3_, 5 mM HEPES, 30 mM Sucrose, 5 mM Trehelose, 10 mM MgCl_2_). Larvae were rinsed with HL-3 to remove debris, central nervous systems were carefully removed by cutting the innervating motor neurons, and the HL-3 without Ca^2+^ solution was replaced with 4μM FM 1-43FX in HL-3 containing 1 mM Ca^2+^ and 90 mM KCl. After 1 min, the FM 1-43FX was removed and larvae were washed five times over 5–10 min with HL-3 without Ca^2+^. Larvae were then fixed for 5 min with 3.7% PFA diluted in HL-3 without Ca^2+^. The fixative was washed off through a series of five washes over 15 min with HL-3 without Ca^2+^ containing 2.5% normal goat serum (Thermo Fisher Scientific). Dissected larvae were unpinned and placed in 1.5 mL centrifuge tubes containing HL-3 without Ca^2+^. Larvae were washed five times with HL-3 without Ca^2+^ over a 10 min period and then incubated with A647 HRP (1:100, diluted in HL-3 without Ca^2+^) for 30 min. Finally, the A647 HRP solution was removed and five washes using HL-3 without Ca^2+^ were performed. Samples were placed on microscope slides, covered with Vectashield mounting medium, and imaged the same day.

### Image acquisition and analysis

6/7 NMJs of body wall segments 3 or 4 were imaged using the 60x oil immersion objective on an Olympus Fluoview 1,000 laser scanning confocal microscope. For each experimental replicate, all genotypes were immunostained using the same reagents. Confocal acquisition settings were obtained for all controls, averaged, and then used for experimental animals. Approximately equal numbers of controls and experimental animals were imaged each day. Each experiment included at least two biological replicates.

Image z-stacks were processed in Fiji (NIH Image J) (Schindelin et al., 2012). Using max projection confocal images, NMJs were outlined and relative fluorescence was calculated by subtracting the background fluorescence from the synaptic fluorescence. All values reported from immunohistochemistry experiments were normalized to the average relative fluorescence of control animals. Bruchpilot (Brp) densities were calculated by manually counting the number of NMJ Brp puncta and dividing by the area of the NMJ. The distance between Brp and GluRIIC was determined by drawing lines through boutons perpendicular to the NMJ branch on z-projected images, generating red-green intensity profiles, and calculating the distance between the maximum peaks for Brp and GluRIIC. Peak distances were calculated for five terminal boutons per NMJ and the mean was used to represent each NMJ.

To measure FM 1-43FX signal intensity, NMJ region of interests were obtained from max projection confocal images. For each z-stack slice, relative fluorescence was calculated by subtracting background fluorescence from synaptic fluorescence. The relative fluorescence value of each slice was averaged and reported as mean NMJ fluorescence intensity.

### RNA isolation and RT-qPCR

Central nervous systems and muscle pelts were dissected from third instar larvae in Roger's Ringer solution and placed into nuclease-free 1.5 mL centrifuge tubes containing 200 μL of RNAlater (Thermo Fisher Scientific). Dissected tissues were stored at −20°C until RNA isolation was performed using the Ambion PureLink RNA Mini Kit (Thermo Fisher Scientific). RNA concentrations were determined using an Implen NanoPhotometer N50. Reverse transcription quantitative polymerase chain reaction (RT-qPCR) was performed using the iTaq Universal SYBR Green One-Step Kit (BioRad). 100 ng of RNA and 50 pmol/μl of cDNA-specific primers were added to each reaction. RT-qPCR was performed using a CFX Connect thermal cycler (BioRad) to obtain cycle threshold or C(t) values. Heat maps were generated using GraphPad Prism (v. 9.3.0) from 2^−(Δ*ΔCt*)^, which was calculated by first subtracting the C(t) value of the target transcript reaction from the C(t) value for GAPDH to obtain ΔC(t) for each transcript. Next, the difference between control and *tsp* mutant ΔC(t)s was calculated to obtain the ΔΔC(t), which was subsequently log transformed. At least three biological replicates including three technical replicates were used for data analysis.

### Electrophysiology

Third instar larvae were dissected on Sylgard-coated coverslips (World Precision Instruments) in ice cold HL-3 containing 0.25 mM Ca^2+^, which was replaced with room temperature HL-3 containing 1.0 mM Ca^2+^ for recordings. Two electrode voltage clamp was performed on muscle six of body wall segments 3 or 4 using electrodes with resistances of 10–30 MΩ filled with 3 M KCl. Muscles were clamped at −60 mV using an Axoclamp 900A amplifier (Molecular Devices). Recordings were collected in pClamp (v. 11.1) and only obtained from muscles if the input resistance was <5 MΩ. Suprathreshold stimuli were delivered to segmental nerves using a suction electrode filled with bath saline and a Grass S88 stimulator with a SIU5 isolation unit (Grass Technologies). Quantal content was calculated by dividing the integrated area of evoked currents by the integrated area of spontaneous currents (eEJC nA ^*^ ms/mEJC nA ^*^ ms) as previously described (Bykhovskaia, 2008). The high frequency stimulation protocol consisted of stimuli administered at 0.2 Hz for 50 s, 20 Hz for 60 s, and 0.2 Hz for 50 s. Paired pulse amplitudes were measured after delivering two each of 10, 20, 50, and 100 Hz pulses with each pair separated by a 20 s intertrial interval. To measure the size of the vesicle pools, dissected larvae were incubated at room temperature for 20 min in freshly prepared 2 μM Bafilomycin in HL-3 containing 1 mM Ca^2+^. After mEJCs were recorded, the segmental nerve was stimulated at 3 Hz for 10 min or at 10 Hz for 5 min. Recordings were digitized with a Digidata 1443 digitizer (Molecular Devices). An approximately equal number of recordings from controls and experimental animals were obtained each day. Data were analyzed in Clampfit (v 11.1, Molecular Devices) and GraphPad Prism (v. 9.3.0).

### Behavior

Third instar larvae were placed onto 1.6% agar plates and allowed to wander for 1 min to remove excess food debris and acclimate to the agar crawling surface. Larvae were then transferred to a 1.6% agar-coated behavioral arena and video recorded for 30 s at 29.97 frames per second with a Canon EOS M50 camera. Each recording was performed on a group of five larvae. Video recordings were analyzed in Fiji with the wrMTrck plugin by Jesper S. Pedersen. Values for distance crawled, average and maximum velocities, and body lengths traveled per second (to normalize for variation in larval body size) were recorded.

### Longevity

Newly enclosed (Day 0) adult flies were collected, separated by sex, and put into vials containing Jazz Mix fly food (Fisher Scientific). Each vial contained 10 adults of the same genotype and sex. Vials were checked daily and deaths were recorded along with the number of days survived. One sample represents one individual (*w*^1118^, *n* = 74; *tsp42Ee*^*G*2619^, *n* = 76; *tsp42Eg*^*MB*08050^, *n* = 64). Survival curves were generated and analyzed in GraphPad Prism (v. 9.3.0).

### Experimental design and statistical analyses

All experiments included at least two biological replicates. Each replicate included an approximately equal number of control and experimental animals. Sample sizes are indicated by data points on graphs. All statistical analyses were performed using GraphPad Prism (v 9.3.0). Unpaired *t* tests were used when comparing one control group to one experimental group. Log-rank (Mantel-Cox) tests were used for survival curve comparison. One-way ANOVAs followed by *post hoc* Tukey's multiple comparisons tests were used for statistical analyses across genotypes for immunocytochemistry experiments when there was more than one control group. *P*-values were adjusted for multiple comparisons. Two-way ANOVAs followed by Dunnett's multiple comparison tests were used to determine if there were differences in evoked currents during high frequency, 3 and 10 Hz stimulation protocols. Statistical significance is denoted on graphs: ^*^ = <0.05, ^**^ = <0.01, ^***^ = <0.001, with error bars representing standard error of the mean (SEM).

## Results

### *Tsp42Ee* and *Tsp42Eg* are CD63 orthologs expressed at the *Drosophila* NMJ

**Figure 1:**
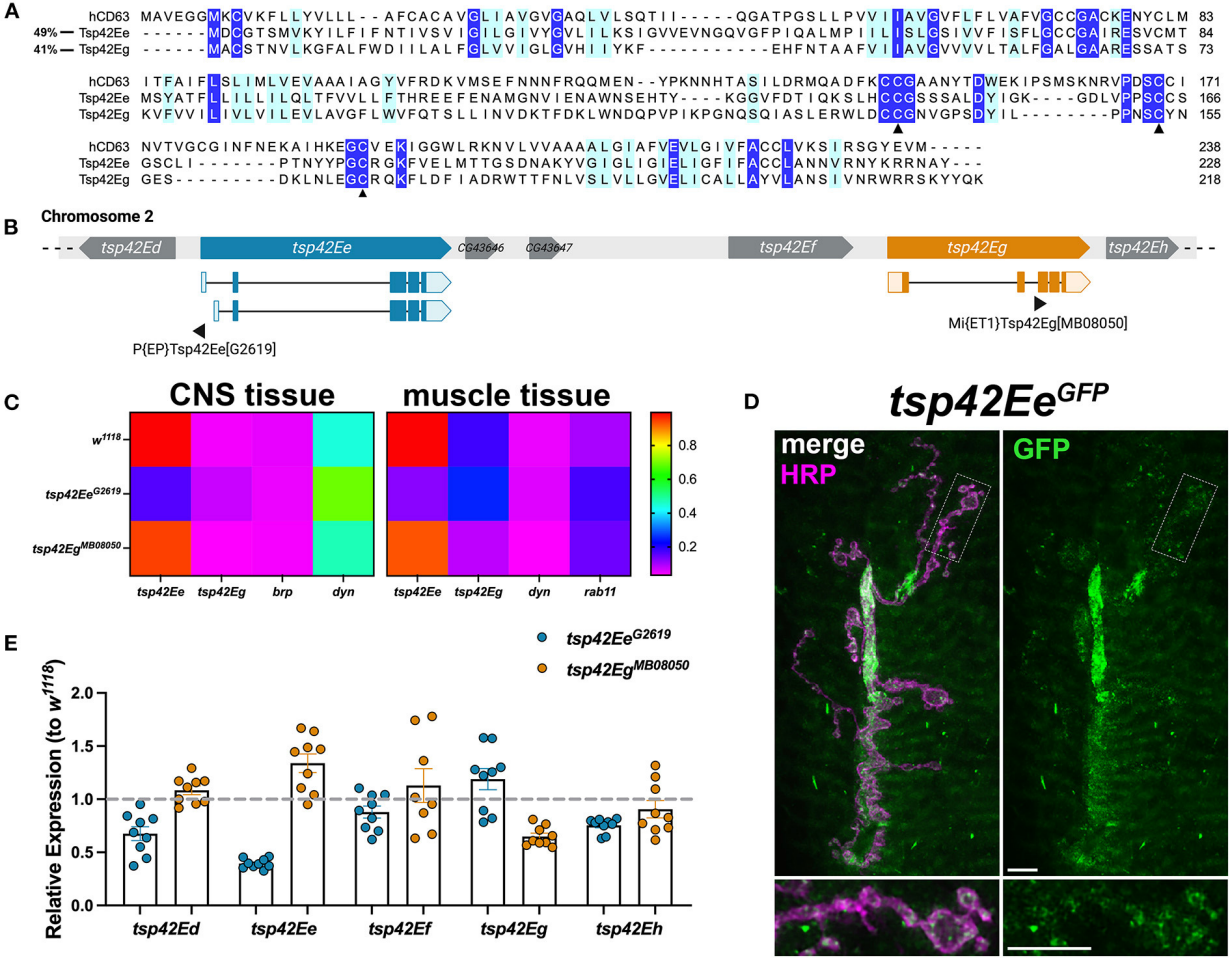
Tsp42Ee and Tsp42Eg are CD63 homologs expressed at the *Drosophila* NMJ. **(A)** Multiple sequence alignment (Clustal Omega) of hCD63, Tsp42Ee, and Tsp42Eg. Identical residues (dark blue) and similar residues as determined by biochemical properties (light blue) are indicated. Arrowheads denote regions of critical motif conservation. **(B)** Arrangement of *tsp* genes (*tsp42Ed*–*tsp42Eh*) on *Drosophila* chromosome 2. Exonic regions of *tsp42Ee* and *tsp42Eg* are mapped with lighter shaded regions denoting 5' and 3' untranslated regions. Sites of transposon insertion for each *tsp* mutant construct are indicated below. **(C)** Heat maps of relative transcript expression in *tsp42Ee*^*G*2619^ and *tsp42Eg*^*MB*08050^ CNS and muscle tissue relative to controls (*w*^1118^). **(D)** Representative confocal image of the 6/7 NMJ showing the localization of GFP-tagged Tsp42Ee (green) in presynaptic terminal boutons (magenta, HRP). Scale bar = 5 μM. **(E)** Histograms of *tsp* transcript expression in *tsp42Ee*^*G*2619^ and *tsp42Eg*^*MB*08050^ whole larvae relative to controls (*w*^1118^; dashed line). Each point represents one technical replicate. Error bars represent SEM.

Tsps are transmembrane proteins that form homophilic and heterophilic complexes to organize membrane microdomains (Termini and Gillette, 2017). There are 33 Tsps in humans (Murru et al., 2018) and 37 in *Drosophila* (Todres et al., 2000) but little is known about their roles at the synapse. Three Tsps are expressed in the motor neuron (Fradkin et al., 2002) and three are expressed in the muscle (flybase.org; Gramates et al., 2022) of the glutamatergic *Drosophila* larval NMJ. To better understand the function of synaptic Tsps, we focused on two previously unexamined Tsps, Tsp42Ee and Tsp42Eg. Both are homologs of CD63, which is best characterized for its interactions with β1-integrin and its roles in cell migration and adhesion (Justo and Jasiulionis, 2021). Tsp42Ee is 24% identical and 49% similar as human CD63 while Tsp42Eg is 26% identical and 41% similar as human CD63 (Figure 1A). Importantly, both Tsp42Ee and Tsp42Eg demonstrate conservation of the canonical CCG motif and two cysteine residues (Figure 1A, arrowheads) located in the EC2 extracellular loop (Seigneuret et al., 2001). The CCG motif and cysteine residues participate in the formation of stabilizing disulfide bridges and thus, are critical for Tsp structure (Kitadokoro et al., 2001). Regions devoid of sequence conservation, especially those in the EC2 extracellular loop, mediate interactions between Tsps and other membrane proteins (Kovalenko et al., 2005).

We used the loss of function mutants, *tsp42Ee*^*G*2619^ and *tsp42Eg*^*MB*08050^, to examine their roles at the synapse. Both alleles result from transposon insertions in the genes (Figure 1B) (Bellen et al., 2011). Using RT-qPCR, we found *tsp42Ee*^*G*2619^ expresses 43.8% of control *tsp42Ee* while *tsp42Eg*^*MB*08050^ expresses 45.5% of *tsp42Eg*. Both mutants are homozygous viable with mean lifespans similar as controls (*w*^1118^ = 73.72 ± 3.35 days, *n* = 74; *tsp42Ee*^*G*2619^ = 70.03 ± 2.94 days, *n* = 76, *p* = 0.41; *tsp42Eg*^*MB*08050^ = 79.59 ± 1.45 days, *n* = 64, *p* = 0.13). However, both *tsp* mutants show significant differences in survival curves relative to controls (*tsp42Ee*^*G*2619^, *p* = 0.0003; *tsp42Eg*^*MB*08050^, *p* = 0.0222; Supplementary Figure 1) indicating that their median survival differs from controls. Therefore, while Tsp42Ee and Tsp42Eg are important for overall survival, Tsps may have functionally redundant physiological roles over the lifespan as previously suggested (Fradkin et al., 2002).

*tsp42Ee* and *tsp42Eg* are expressed in the central nervous system (CNS) and postsynaptic muscle cells in *w*^1118^ controls (Figure 1C). *tsp42Ee* is highly expressed in both CNS and muscle tissue relative to *bruchpilot* (*brp*) and *dynamin* (*dyn*) (Figure 1C), which encode an active zone scaffold protein (Wagh et al., 2006) and a GTPase required for endocytosis (McMahon and Boucrot, 2011), respectively. Tsp42Ee is found in synaptic boutons at the NMJ as indicated by expression of *tsp42Ee*^*CC*01420^, which encodes a Tsp42Ee GFP fusion protein (Figure 1D). *tsp42Eg* is more highly expressed in postsynaptic muscle than CNS in *w*^1118^ controls (Figure 1C). It is expressed in muscle cells at slightly higher levels than *rab11*, which encodes a GTPase that facilitates vesicle trafficking from recycling endosomes to the plasma membrane (Ng and Tang, 2008). *brp, dyn*, and *rab11* transcripts were expressed similarly in *w*^1118^ controls and *tsp* mutants (Supplementary Figure 2).

*tsp42Ee* and *tsp42Eg* are found within a cluster of 18 *tsp* genes, *tsp42Ea*-*tsp42Er*, on the second chromosome (Figure 1B). Given their proximity in the genome, we used RT-qPCR to assess the transcripts encoded by the *tsps* adjacent to *tsp42Ee* and *tsp42Eg* in *tsp42Ee*^*G*2619^ and *tsp42Eg*^*MB*08050^ mutants (Figure 1E). We used whole larvae for these analyses because there are no reports of *tsp42Ed* or *tsp42Ef* expression in the CNS or postsynaptic muscle. While *tsp42Ed, tsp42Ee, tsp42Ef* , and *tsp42Eh* were similar as controls in *tsp42Eg*^*MB*08050^ mutants, *tsp42Ed* and *tsp42Eh* were slightly lower than controls in *tsp42Ee*^*G*2619^ mutants. *tsp42Ed* is expressed in the circulatory and digestive systems and *tsp42Eh* is expressed in the integumentary system and more highly in adult than larval muscles (flybase.org). Therefore, we began by investigating the function of Tsps at *tsp42Ee*^*G*2619^ and *tsp42Eg*^*MB*08050^ mutant synapses.

### Tsp42Ee and Tsp42Eg promote neurotransmission by facilitating neurotransmitter release

**Figure 2:**
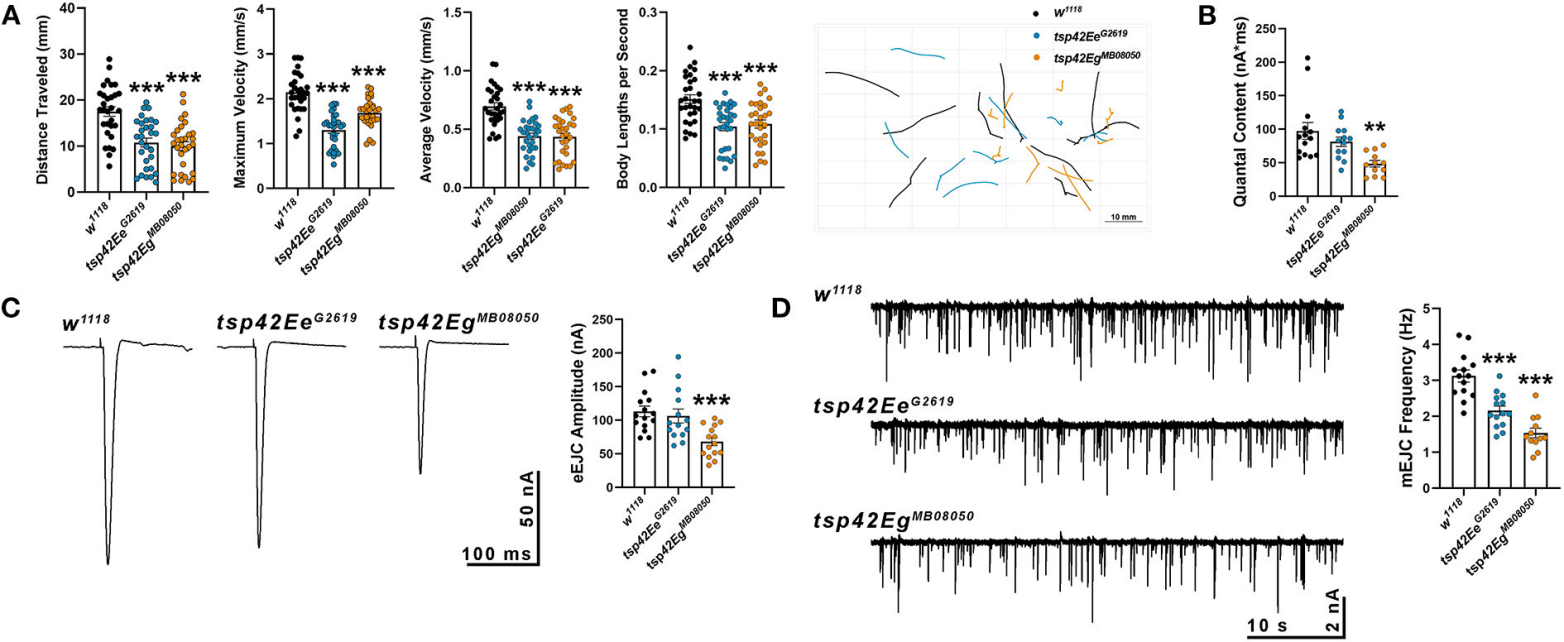
Tetraspanin function is important for proper vesicle release and locomotion. **(A)** wrMTrck quantification of larval crawling behavior on an agar arena for 30 s. Distance traveled, maximum velocity, average velocity, and crawling velocity normalized to body size (body lengths per second) were significantly reduced in both *tsp42Ee* (*p* < 0.0001) and *tsp42Eg* mutants (*p* < 0.0001). **(B)** Quantal content is significantly reduced in *tsp42Eg*^*MB*08050^ (*p* = 0.0022) but not *tsp42Ee*^*G*2619^ (*p* = 0.28) mutants. **(C)** Representative traces (left) and quantification (right) show reduced eEJC amplitudes in *tsp42Eg*^*MB*08050^ (*p* < 0.0001) but not *tsp42Ee*^*G*2619^ (*p* = 0.60) mutants. **(D)** Representative mEJC traces (left) from larval body wall muscle 6. Quantification (right) of mEJC frequency indicates both *tsp42Ee*^*G*2619^ (*p* = 0.0001) and *tsp42Eg*^*MB*08050^ (*p* < 0.0001) mutants exhibit significant reductions in mEJC frequency. Error bars represent SEM. Unpaired *t* tests were used for all statistical comparisons.

Synaptic function in *tsp* mutants was assessed by examining larval crawling behavior and recording miniature and evoked endplate junctional currents (mEJCs and eEJCs, respectively). Larval locomotor behavior relies on both central nervous system central pattern generators and peripheral motor neurons (Heckscher et al., 2012; Gjorgjieva et al., 2013). Although there is not always a correlation between NMJ function and movement, there is a positive correlation between the frequency and duration of motor neuron activity and contractile force of postsynaptic muscles (Ormerod et al., 2022). *tsp42Ee*^*G*2619^ and *tsp42Eg*^*MB*08050^ mutants showed impaired movement as evidenced by reductions in maximum and average larval crawling velocity. These resulted in decreased total distance traveled (Figure 2A). To determine whether the observed movement deficits correlated with synaptic function, we recorded mEJCs and eEJCs from muscle six of third instar larva using two electrode voltage clamp. *tsp42Eg*^*MB*08050^ but not *tsp42Ee*^*G*2619^ mutants exhibited reduced eEJC amplitudes compared to *w*^1118^ controls (Figure 2C) producing reduced quantal content compared with controls (Figure 2B). Both *tsp* mutants showed reductions in mEJC frequency compared to controls but there were no differences in mEJC amplitudes (Figure 2D; *w*^1118^ = 1.23 nA, *n* = 14; *tsp42Ee*^*G*2619^ = 1.16 nA, *n* = 14, *p* = 0.70; *tsp42Eg*^*MB*08050^ = 1.16 nA, *n* = 12, *p* = 0.66). The reduction in mEJC frequency indicates that both *tsp* mutants may possess fewer functional active zones.

**Figure 3:**
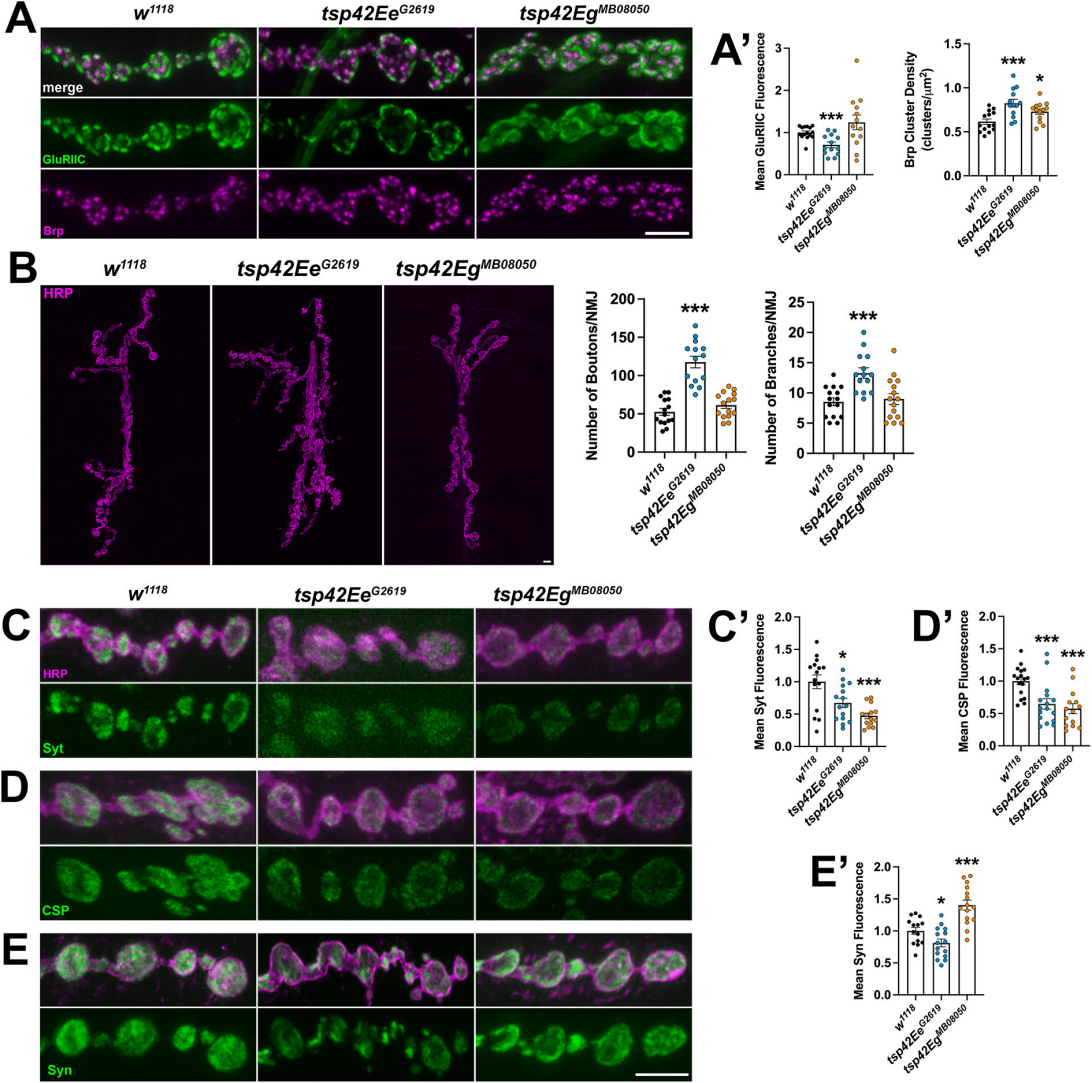
Tsp42Ee and Tsp42Eg are important for active zone organization and vesicle-associated protein localization. **(A)** Panels show representative images of control (*w*^1118^) and *tsp* mutant boutons immunolabeled for the active zone marker, Brp (magenta), and postsynaptic glutamate receptor subunit, GluRIIC (green). **(A')** Quantification of mean Brp puncta density and fluorescence intensity of GluRIIC. Brp density is significantly increased in both *tsp42Ee*^*G*2619^ (*p* = 0.0006) and *tsp42Eg*^*MB*08050^ (*p* = 0.0142) mutants. GluRIIC fluorescence is significantly reduced in *tsp42Ee*^*G*2619^ mutants (*p* = 0.0005). **(B)** Confocal images of HRP-labeled 6/7 NMJ's (left) and quantification of bouton and branch number (right). Boutons (*p* < 0.0001) and branch numbers (*p* = 0.0001) were significantly increased in *tsp42Ee*^*G*2619^ mutants. **(C–E)** Representative confocal images showing HRP-labeled neurons (magenta) and the localization of Syt **(C)**, CSP **(D)**, or Syn **(E)** (green). Right bar graphs show quantification of immunofluorescence normalized to *w*^1118^ controls. Mean Syt fluorescence **(C')** is significantly reduced in *tsp42Ee*^*G*2619^ (*p* = 0.0168) and *tsp42Eg*^*MB*08050^ (*p* = 0.0001) mutants. Mean CSP fluorescence **(D')** is significantly reduced in *tsp42Ee*^*G*2619^ (*p* = 0.0010) and *tsp42Eg*^*MB*08050^ (*p* < 0.0001) mutants. Mean Syn fluorescence **(E')** is significantly reduced in *tsp42Ee*^*G*2619^ mutants (*p* = 0.0295) but increased in *tsp42Eg*^*MB*08050^ mutants (*p* = 0.0003). Scale bars = 5 μM. Errors bars represent SEM. Unpaired *t* tests were used for all statistical comparisons.

Each synaptic bouton contains several active zones, which include the scaffold protein Brp (Wagh et al., 2006). We quantified the density of active zones as indicated by Brp and found that both *tsp* mutants showed an increase in density of active zones compared with controls (Figures 3A,A'). Active zones are closely apposed to postsynaptic glutamate receptors and this apposition is important for the efficiency of neurotransmission. There were no differences in apposition as indicated by the distances between Brp and the essential postsynaptic glutamate receptor subunit, GluRIIC, in either *tsp* mutant (data not shown). There was, however, a decrease in GluRIIC fluorescence intensity in *tsp42Ee*^*G*2619^ mutants compared to controls (Figures 3A,A').

The reduction in mEJC frequency in *tsp* mutants also led us to examine synaptic morphology and synaptic proteins important for vesicle release. We examined gross morphology of motor neurons using α-Horseradish peroxidase (HRP), which recognizes neuronal N-glycans (Parkinson et al., 2013), to label neuronal membranes. Motor neurons contain presynaptic boutons arranged within branched arbors (Menon et al., 2013). While *tsp42Eg*^*MB*08050^ mutants were morphologically similar as controls, *tsp42Ee*^*G*2619^ mutants exhibited overgrown motor neurons characterized by increased numbers of branches and boutons (Figure 3B).

We next examined several additional presynaptic proteins. Syt binds Ca^2+^ to enable SNARE complex formation thereby facilitating exocytosis of presynaptic vesicles (Hackett and Ueda, 2015) and CSP is a vesicle-associated protein chaperone (Gundersen, 2020). Synaptic levels of both Syt and CSP were reduced in *tsp* mutants compared with *w*^1118^ controls (Figures 3C,C',D,D'). Similarly, Syn, a protein that tethers the reserve pool of vesicles to the actin cytoskeleton (Hackett and Ueda, 2015), was reduced in *tsp42Ee*^*G*2619^ mutants but increased in *tsp42Eg*^*MB*08050^ mutants (Figures 3E,E'). The vesicular glutamate transporter, vGLUT, however, was similar in mutants and controls (Supplementary Figure 3). These data indicate that the impaired synaptic function in *tsp* mutants may be due to a reduction in the release probability of vesicles. We investigated this possibility by performing paired pulse recordings at *tsp* mutant NMJs. Increases in paired pulse ratios are correlated with a decrease in release probability (Regehr, 2012). There were no significant differences in paired pulse ratios in *tsp* mutants at interstimulus intervals of 10, 20, 50, or 100 ms (Supplementary Figure 4) indicating that intracellular Ca^2+^ dynamics at *tsp* mutant active zones are unaffected.

### Tsp42Ee and Tsp42Eg differentially regulate synaptic vesicle pools to restrict endocytosis

**Figure 4:**
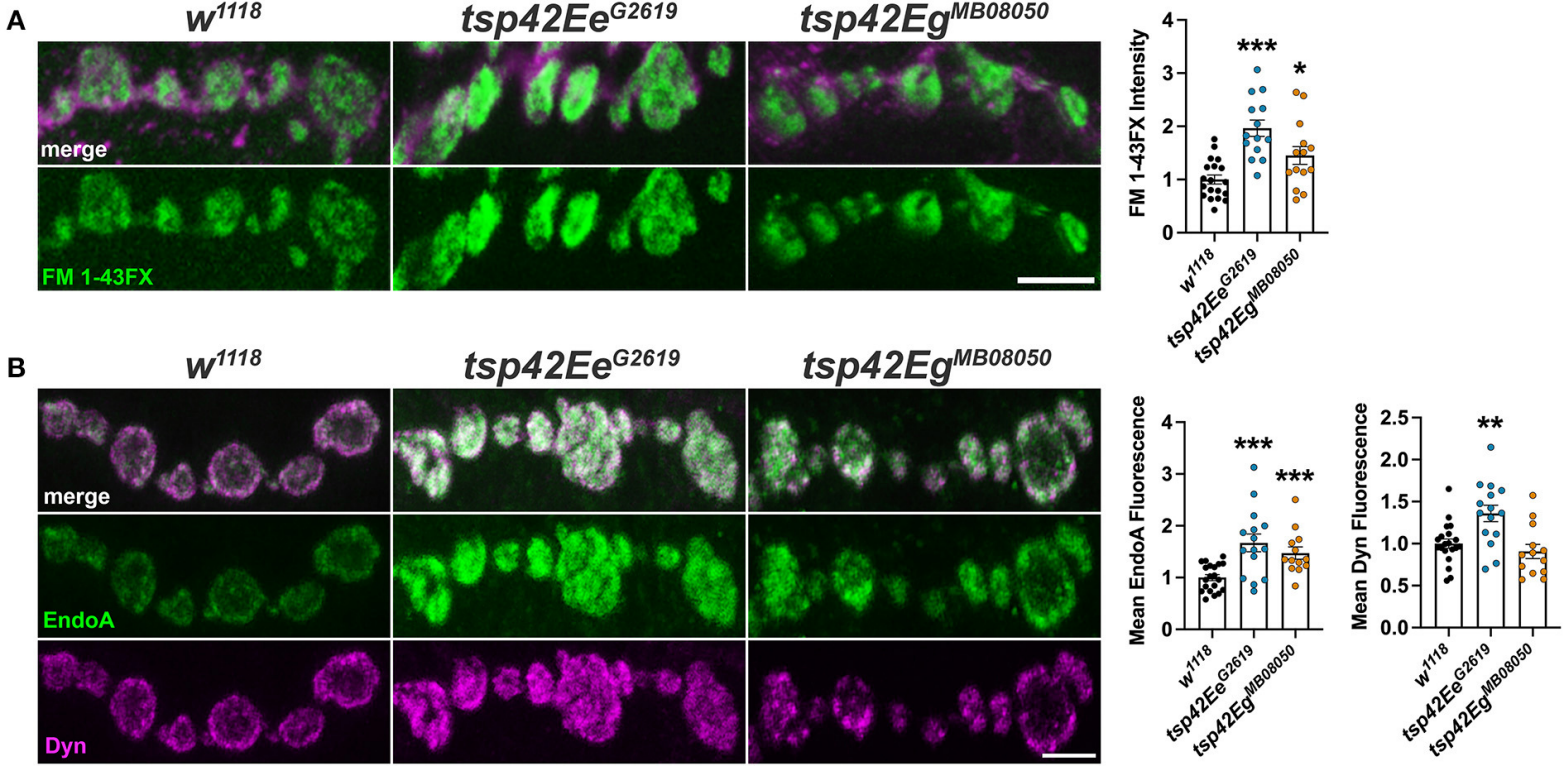
Tsps negatively regulate endocytosis and endocytic protein localization. **(A)** Representative confocal images showing relative rates of endocytosis in HL-3 + 1.0 mM Ca^2+^ as measured by FM 1-43FX immunofluorescence at 6/7 terminal boutons (left). Quantification of FM 1-43FX fluorescence intensity relative to *w*^1118^ controls (right). Both *tsp42Ee*^*G*2619^ and *tsp42Eg*^*MB*08050^ mutants show significantly higher endocytosis (*p* < 0.0001 and *p* = 0.0144, respectively). **(B)** Representative images of terminal boutons (left) immunolabeled for EndoA (green) and Dyn (magenta) and quantification of mean immunofluorescence (right). EndoA fluorescence is significantly increased in both *tsp42Ee*^*G*2619^ (*p* = 0.0002) and *tsp42Eg*^*MB*08050^ mutants (*p* = 0.0004). Mean Dyn fluorescence is significantly increased in *tsp42Ee*^*G*2619^ mutants (*p* = 0.0018). Scale bars = 5 μM. Errors bars represent SEM. Unpaired *t* tests were used for all statistical comparisons.

Altered endocytosis may contribute to reductions in evoked and spontaneous neurotransmission in *tsp* mutants. Therefore, we assessed endocytosis using the lipophilic dye, FM 1–43FX, to label newly endocytosed synaptic vesicles (Verstreken et al., 2008) after 1 min stimulation with 1.0 mM Ca^2+^ and 90 mM KCl. Surprisingly, both *tsp* mutants exhibited an increase in endocytosis compared with controls (Figure 4A). To ensure mutations in *tsps* do not affect the affinity of FM 1-43FX for the membrane, we examined FM 1-43FX intensities in the absence of stimulation and found no differences between controls and *tsp* mutants (Supplementary Figure 5).

Endocytosis requires Endophilin A (EndoA) to facilitate membrane invagination and recruit Dyn (Kjaerulff et al., 2011). EndoA is recruited to perisynaptic membranes by the endocytic scaffold, Dap160/Intersectin, which directly interacts with Eps15 (Koh et al., 2007). EndoA was increased in both *tsp* mutants compared to controls while Dyn was increased in *tsp42Ee*^*G*2619^ but not *tsp42Eg*^*MB*08050^ mutants (Figure 4B). Conversely, there were no differences in synaptic levels of Dap160 or Eps15 in *tsp* mutants (data not shown). Thus, the increase in endocytosis in *tsp* mutants may be partly explained by increases in synaptic EndoA and/or Dyn.

In addition to the proper localization of endocytic proteins, neurotransmission relies on the coordinated mobilization and trafficking of vesicles from the reserve (RP), readily releasable (RRP), and recycling pools (Augustine et al., 1999; Alabi and Tsien, 2012). Vesicles in the RRP are docked at presynaptic active zone release sites and, therefore, are the first to be released upon stimulation (Rosenmund and Stevens, 1996; Hoopmann et al., 2010). The RP, however, is mobilized upon high frequency stimulation to replenish the RRP (Pieribone et al., 1995; Zhang and Augustine, 2021). To sustain rapid vesicle release at the synapse, recycling of synaptic vesicles through the endocytic and endosomal sorting pathways must occur (Hoopmann et al., 2010; Saheki and De Camilli, 2012). Thus, disruptions in vesicle trafficking through endosomal pathways and synaptic vesicle pools may alter endocytosis and compromise neurotransmitter release.

**Figure 5:**
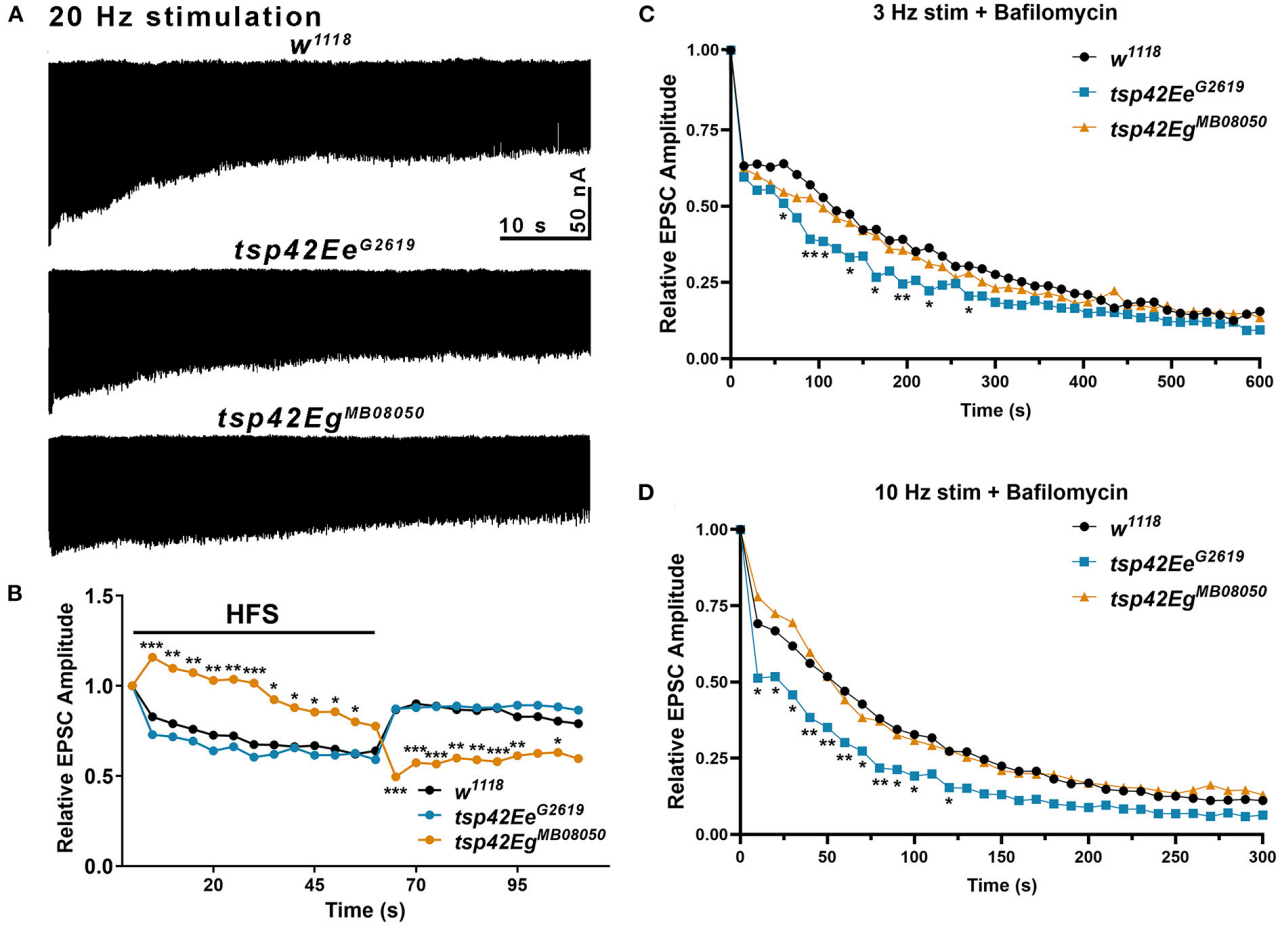
Tsp42Ee and Tsp42Eg differentially affect evoked responses to high frequency stimulation and synaptic vesicle pools. **(A)** Representative recordings from animals during 20 Hz stimulation in HL-3 + 1.0 mM Ca^2+^. **(B)** Relative EPSC amplitudes (to the first stimulus) over time during both 20 Hz stimulation (first 60 s) and the recovery period (final 50 s). *Tsp42Eg*^*MB*08050^ mutants showed increased EPSC amplitudes during the first 55 s of 20 Hz stimulation but decreased amplitudes during the recovery period as determined by a two-way ANOVA (*F*_(39, 858)_ = 15.27, *p* < 0.0001) followed by *post hoc* Dunnett's multiple comparisons tests. **(C)** Relative EPSC amplitudes over time during 3 Hz stimulation in HL-3 containing 2 μm Bafilomycin and 1.0 mM Ca^2+^. *Tsp42Ee*^*G*2619^ mutants showed significant reductions in EPSC amplitudes at 60, 90, 105, 135, 165, 195, 225, and 270 s as determined by a two-way ANOVA (*F*_(29, 1160)_ = 71.38, *p* < 0.0001) followed by *post hoc* Dunnett's multiple comparisons tests. **(D)** Relative EPSC amplitudes over time during 10 Hz stimulation in HL-3 containing 2 μm Bafilomycin and 1.0 mM Ca^2+^. *Tsp42Ee*^*G*2619^ mutants showed significant reductions in EPSC amplitudes at 10–100 and 120 s as determined by a two-way ANOVA (*F*_(28, 840)_ = 86.10, *p* < 0.0001) followed by *post hoc* Dunnett's multiple comparisons tests.

To determine if *tsp* mutants exhibited altered vesicle pool dynamics, we assessed evoked responses induced by several stimulation paradigms. First, we examined both clathrin-mediated and activity-dependent bulk endocytosis by recording eEJCs in 1.0 mM Ca^2+^ during and after high frequency stimulation. This stimulation first utilizes the RRP of vesicles, then mobilizes the RP of vesicles, and measures recycling of newly endocytosed synaptic vesicles (Delgado et al., 2000; Long et al., 2010; Müller et al., 2012). eEJC amplitudes were assessed at 20 Hz stimulation for 60 s followed by a recovery period of 0.2 Hz stimulation for a 50 s (Long et al., 2010). During high frequency stimulation, controls show a rapid reduction in eEJC amplitudes followed by increased eEJC amplitudes during the post-stimulation recovery period when the RRP of vesicles is replenished. *tsp42Ee*^*G*2619^ mutant eEJCs were similar as *w*^1118^ controls at all time points (Figures 5A,B). *tsp42Eg*^*MB*08050^ mutants, however, exhibited potentiated eEJCs for the first 30 s of high frequency stimulation followed by a gradual decline in eEJCs. The increase in eEJC amplitudes during high frequency stimulation in *tsp42Eg*^*MB*08050^ mutants is consistent with the increase in FM 1-43FX uptake (Figure 4A). During the recovery period, however, eEJCs were reduced in *tsp42Eg*^*MB*08050^ mutants indicating vesicle recycling is impaired. Therefore, even though there is increased endocytosis in *tsp42Eg*^*MB*08050^ mutants, the endocytosed vesicles are recycled slower than controls.

To assess whether the increase in endocytosis occurs in *tsp* mutants because of increased vesicle pool sizes, we used Bafilomycin A1, which inhibits vesicular H^+^ pumps to block glutamate uptake into vesicles (Cavelier and Attwell, 2007). In the presence of Bafilomycin, newly endocytosed vesicles will not be refilled with glutamate and eEJC amplitudes will diminish over time as vesicles that lack glutamate are released. Low frequency, 3 Hz stimulation relies on the RRP and recycling pools of vesicles. Higher frequency, 10 Hz stimulation mobilizes the RP of vesicles (Delgado et al., 2000). We examined eEJC amplitudes after 20 min incubation with Bafilomycin in 1.0 mM Ca^2+^ during 3 or 10 Hz stimulation. The initial decline in eEJC amplitudes at both 3 and 10 Hz was more pronounced in *tsp42Ee*^*G*2619^ mutants compared with controls (Figures 5C,D) suggesting these animals possess smaller vesicle pools. There were no differences in *tsp42Eg*^*MB*08050^ mutants at any time point during either 3 or 10 Hz stimulation. Collectively, these data suggest that the increase in endocytosis at *tsp* mutant synapses occurs through different mechanisms.

### Tsps regulate synaptic cytoskeleton structure and membrane lipid composition

**Figure 6:**
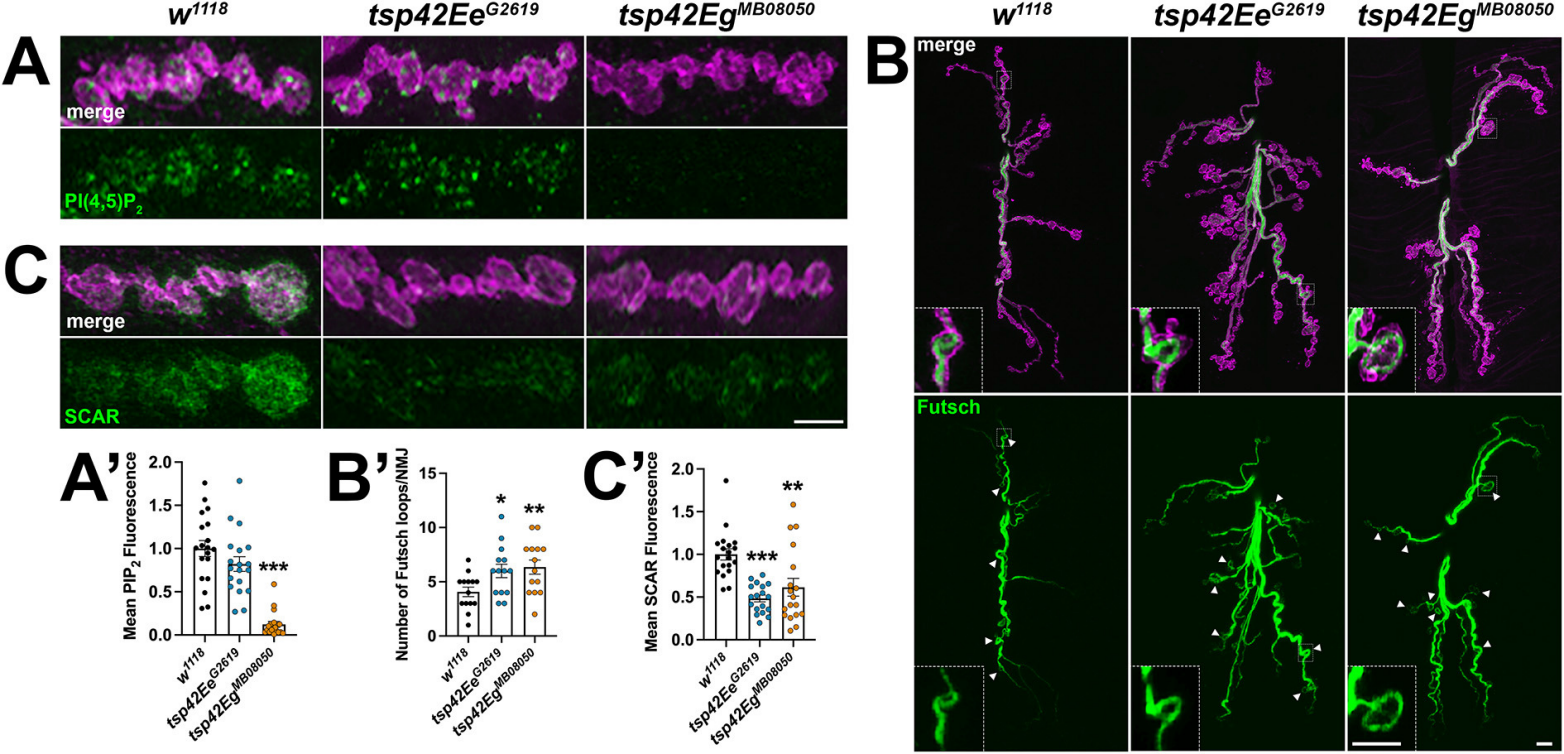
Tsp42Ee and Tsp42Eg regulate synaptic membrane lipids and cytoskeletal structures. **(A,C)** Panels showing terminal boutons (HRP, magenta) immunolabeled for PI(4,5)P_2_ (A, green) or SCAR (C, green). **(A')** Quantification of mean PI(4,5)P_2_ fluorescence intensity relative to *w*^1118^ controls. PI(4,5)P_2_ fluorescence is decreased at *tsp42Eg*^*MB*08050^ mutant synapses (*p* < 0.0001). **(B)** Confocal images of 6/7 NMJs (HRP, magenta) immunolabeled for Futsch (green), a microtubule-binding protein. Arrowheads indicate Futsch-labeled looped microtubules with inset panels showing details of a representative Futsch loop. **(B')** Histograms display numbers of Futsch-positive loops void of punctate signal at control and *tsp* mutant NMJs. Futsch loops are significantly increased in *tsp42Ee*^*G*2619^ (*p* = 0.0174) and *tsp42Eg*^*MB*08050^ mutants (*p* = 0.0073). **(C')** Quantification of mean SCAR fluorescence intensity relative to *w*^1118^ controls. SCAR fluorescence intensity is significantly decreased in both *tsp42Ee*^*G*2619^ and *tsp42Eg*^*MB*08050^ mutants (*p* < 0.0001 and *p* = 0.0032, respectively). Scale bars = 5 μM. Errors bars represent SEM. Unpaired *t* tests were used for all statistical comparisons.

The recruitment and assembly of endocytic machinery is influenced by membrane lipid composition (Sun et al., 2007). Specifically, phosphatidylinositol-4,5-bisphosphate [PI(4,5)P_2_] organizes into microdomains and regulates endocytosis, vesicle trafficking, and NMJ growth by interacting with cytoskeleton-binding and synaptic vesicle-associated proteins (Cremona et al., 1999; Khuong et al., 2010; Mandal, 2020). Thus, Tsps may negatively regulate endocytosis (Figure 4A) and endocytic protein localization (Figure 4B) by regulating synaptic PI(4,5)P_2_ distribution. Notably, CD63 directly interacts with Syntenin-1, a high affinity PI(4,5)P_2_ binding protein (Mortier et al., 2005; Latysheva et al., 2006). There was a marked reduction in PI(4,5)P_2_ at *tsp42Eg*^*MB*08050^ mutant synapses while *tsp42Ee*^*G*2619^ mutants showed no change in synaptic PI(4,5)P_2_ levels (Figures 6A,A'). These results, however, do not explain why endocytosis is increased in *tsp* mutants as PI(4,5)P_2_ is important for both clathrin-mediated and activity-dependent bulk endocytosis (Sun et al., 2007; Li et al., 2020). One possibility is that Tsps recruit cytoskeletal proteins to sites of endocytosis and regulate vesicle trafficking independent of PI(4,5)P_2_ microdomains.

Microtubules and F-actin are foundational building blocks of the synaptic cytoskeleton. Their dynamics regulate active zone organization, neurotransmission, and vesicle transport (Roos et al., 2000; Lepicard et al., 2014; Piriya Ananda Babu et al., 2020). Furthermore, actin assembly at sites of endocytosis facilitates the mechanics of membrane invagination and endocytic vesicle trafficking (Smythe and Ayscough, 2006; Liu et al., 2009). The highly dynamic microtubule cytoskeleton is regulated by covalent modifications such as tubulin acetylation, polyglutamylation, and detyrosination that either promote microtubule polymerization or depolymerization. Specifically, α-tubulin acetylation at Lys-40 confers stability to microtubule polymers (Li and Yang, 2015) and disruptions in synaptic microtubule integrity lead to defective synaptic vesicle anchoring and neurotransmitter release (Piriya Ananda Babu et al., 2020). No significant differences in synaptic acetylated tubulin levels were observed in *tsp* mutants compared with *w*^1118^ controls (data not shown). Thus, alterations in microtubule stability cannot explain the endo/exocytic dysregulation observed at *tsp* mutant NMJs.

The microtubule-binding protein, Futsch, which is the MAP1B homolog, colocalizes with the microtubule cytoskeleton and is required for normal glutamate release at the *Drosophila* NMJ (Lepicard et al., 2014). During periods of synaptic growth, microtubules adopt looped structures that are stabilized by association with Futsch. In contrast, Futsch does not associate with unbundled microtubules found in static boutons (Roos et al., 2000; Ruiz-Canada et al., 2004; Miech et al., 2008). The presence of Futsch loops in synaptic boutons can be used to indicate sites of active growth and cytoskeletal rearrangement (Sarthi and Elefant, 2011). We immunostained for Futsch at 6/7 NMJs and found that both *tsp* mutant synapses have increased Futsch-positive loops (Figures 6B,B') indicating that *tsp* mutant synapses have more dynamic microtubule rearrangements than controls. This result is consistent with the increase in active zone density in *tsp* mutants (Figures 3A,A') as previous findings directly implicate Futsch in the anchoring of active zone components to the microtubule cytoskeleton (Lepicard et al., 2014). These results, however, fail to explain our finding that, while *tsp* mutants have more active zones, some active zones don't function properly during spontaneous or evoked neurotransmission.

We next examined the synaptic localization of two F-actin regulators, Wiskott-Aldrich syndrome protein (WASp) and SCAR. WASp and the WASp family verprolin-homologous (WAVE) protein homolog, SCAR, regulate Arp2/3-dependent actin branching by integrating intracellular signaling inputs in *Drosophila* (Machesky et al., 1999; Ben-Yaacov et al., 2001; Zallen et al., 2002; Stradal et al., 2004). Actin branching is important for maintaining both overall synaptic morphology and the local formation of synaptic actin patches at sites of endocytosis. WASp, through interactions with Dap160, is a regulator of active zone assembly and endocytic function at the *Drosophila* NMJ (Del Signore et al., 2021). We examined WASp at *tsp* mutant synapses and observed no differences in the synaptic levels of WASp in either *tsp42Ee*^*G*2619^ or *tsp42Eg*^*MB*08050^ mutants compared to controls (data not shown). Similarly, the WASp-dependent actin regulator, Nervous Wreck (Nwk) (Coyle et al., 2004) was unchanged at either *tsp* mutant synapse (data not shown). Both *tsp* mutants, however, exhibited decreased levels of SCAR at the synapse (Figures 6C,C'). Upon Rac1 signaling, SCAR induces Arp2/3 activity and subsequent remodeling of the actin cytoskeleton during synaptic development and plasticity (Zallen et al., 2002; Schenck et al., 2004). These results suggest that Tsp42Ee and Tsp42Eg influence synaptic levels of SCAR, but not WASp, to regulate actin branching. These results also suggest that, despite reduced PI(4,5)P_2_ and SCAR in *tsp42Eg*^*MB*08050^ mutants and reduced SCAR in *tsp42Ee*^*G*2619^ mutants, there may be sufficient WASp at the synapse to promote Arp2/3-dependent branching that facilitates increased endocytosis in these animals.

### Expression of human CD63 at the *Drosophila* NMJ attenuates endocytosis

**Figure 7:**
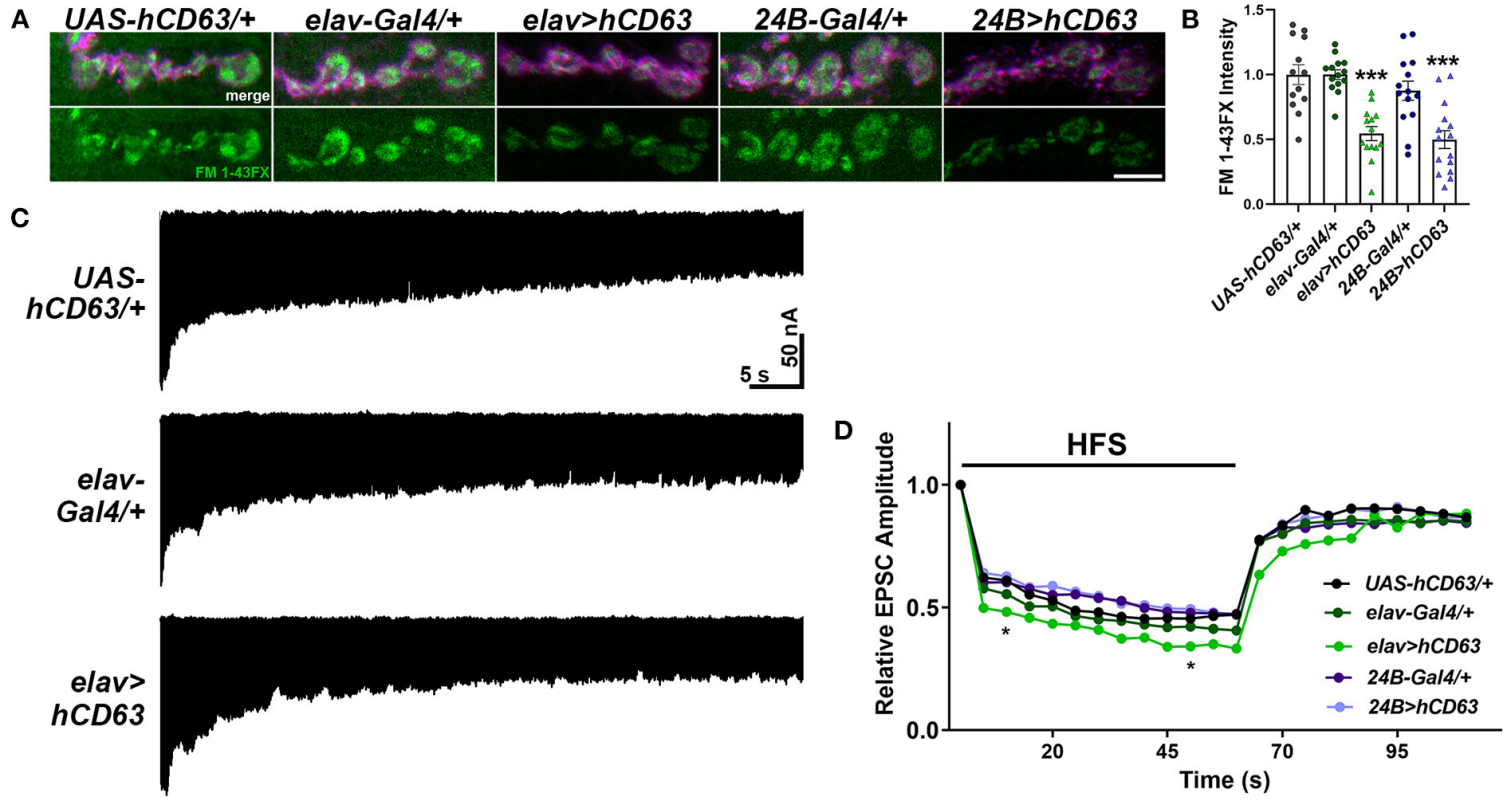
Expression of human CD63 in *Drosophila* larval neurons impairs endocytosis. **(A)** Representative confocal images showing relative rates of endocytosis in HL-3 + 1.0 mM Ca^2+^ as measured by FM 1-43FX immunofluorescence in 6/7 terminal boutons of genotypes as indicated. **(B)** Quantification of FM 1-43FX fluorescence intensity relative to the *UAS-hCD63/*+ outcrossed controls. FM 1-43FX fluorescence is significantly reduced when hCD63 is expressed in neurons (*elav*>*hCD63*; *F*_(2, 38)_ = 21.18, *p* < 0.0001, one-way ANOVA) and in muscle (*24B*>*hCD63*; *F*_(2, 39)_ = 12.97, *p* < 0.0001, one-way ANOVA). **(C)** Representative recordings from animals during 20 Hz stimulation in HL-3 + 1.0 mM Ca^2+^. **(D)** Relative EPSC amplitudes over time during both 20 Hz stimulation (first 60s) and the recovery period (final 50s). Expressing human CD63 (hCD63) in neurons using the *elav-Gal4* driver resulted in a significant decrease in EPSC amplitudes at 10 and 50 s as determined by a two-way ANOVA (*F*_(53, 1166)_ = 25.37, *p* < 0.0001) followed by *post hoc* Dunnett's multiple comparisons tests.

Tsp42Ee and Tsp42Eg are homologs of human CD63 (Figure 1A). CD63 was the first characterized Tsp and is expressed in all cell types. It is localized to the plasma membrane but is enriched on internal membranes including late endosomes and lysosomes (Pols and Klumperman, 2009). The described function of CD63 in neurons is largely limited to its role in the trafficking and biogenesis of exosomes (Andreu and Yanez-Mo, 2014). To investigate the contribution of CD63 to the synaptic vesicle cycle, we expressed human CD63 (hCD63) in neurons using the *elav-Gal4* driver or in postsynaptic muscle using the *24B-Gal4* driver. Expression of hCD63 in either neurons or muscle decreased endocytosis as evidenced by reduced internalization of FM 1-43FX dye (Figures 7A,B). Similarly, there were reductions in EPSC amplitudes at 10 and 50 s after administering 20 Hz high frequency stimulation when hCD63 was expressed in neurons but not in postsynaptic muscle cells (Figures 7C,D). There were no differences in EPSC amplitudes during the recovery period. There were also no differences in eEJC amplitudes, quantal content, mEJC amplitudes, or mEJC frequencies in animals expressing hCD63 in neurons or muscle (data not shown). These data indicate that hCD63, like Tsp42Ee and Tsp42Eg (Figure 4A), restricts endocytosis and may be functionally redundant with Tsp42Ee and Tsp42Eg.

## Discussion

Our findings uncover novel synaptic roles for the *Drosophila* CD63 orthologs, Tsp42Ee and Tsp42Eg, and highlight their shared and unique functions. Both Tsps facilitate basal neurotransmitter release and locomotor output (Figure 2) by regulating synaptic vesicle pool dynamics (Figure 5). Tsp42Ee and Tsp42Eg also promote synaptic localization of the vesicle-associated proteins Syt and CSP (Figures 3C,D) but restrict the cytoskeletal proteins Futsch and SCAR (Figures 6B,C). Finally, we find that Tsp42Ee and Tsp42Eg both negatively regulate endocytosis (Figure 4A). Given that Tsps are organizational hubs (Stipp et al., 2003; Charrin et al., 2014), loss of *tsp42Ee* or *tsp42Eg* function likely affects the synaptic and membrane-specific localization of additional neuronal proteins. Thus, the unique combination of synaptic perturbations in *tsp42Ee*^*G*2619^ and *tsp42Eg*^*MB*08050^ mutants may produce some of the unique phenotypes we observed.

Both *tsp42Ee*^*G*2619^ and *tsp42Eg*^*MB*08050^ mutants exhibited enhanced synaptic endocytosis (Figure 4A) and, in support of functionally redundant roles for Tsps (Fradkin et al., 2002), both *tsp42Ee* and *tsp42Eg* are expressed in presynaptic motor neurons and postsynaptic muscles (Figure 1C) of the NMJ. *tsp42Ee*^*G*2619^ and *tsp42Eg*^*MB*08050^ mutants differed, however, in their evoked responses and in synaptic levels of GluRIIC, Syn, Dyn, and PI(4,5)P_2_ (Figures 3A,E, 4B, 6A). Our findings are consistent with previous studies establishing distinct roles for Tsps as the mammalian Tsps, TSPAN5, TSPAN6, and TSPAN7 also perform different hippocampal functions. While knock down of TSPAN5 does not affect mEPSC amplitude, mEPSC frequency, or evoked amplitudes (Moretto et al., 2019), knock down of postsynaptic TSPAN7 attenuates each of these (Bassani et al., 2012). The AMPA receptor subunits GluA1 and GluA2/3 are significantly reduced in TSPAN7 knock down hippocampal pyramidal neuron cultures (Moretto et al., 2019) but unchanged in *Tspan6* knock out synaptosomes (Salas et al., 2017). Similarly, we observed a significant decrease in GluRIIC and Syn in *tsp42Ee*^*G*2619^ but *tsp42Eg*^*MB*08050^ mutants exhibited no change in GluRIIC and increased Syn (Figures 3A,E). The loss of Syn could account for the reduction in total vesicles in *tsp42Ee*^*G*2619^ mutant NMJs (Figures 5C,D). Mouse Syn triple knock outs (Fornasiero et al., 2012) and *Drosophila syn* knock outs (Akbergenova and Bykhovskaia, 2010) exhibit reductions in the total number of synaptic vesicles. Indeed, *tsp42Ee*^*G*2619^ mutant responses to 10 Hz stimulation in the presence of Bafilomycin mirror that of *syn* knock outs (Akbergenova and Bykhovskaia, 2010). The RRP is unaffected in Syn triple knock outs (Fornasiero et al., 2012) and this may be why, similar as *tsp42Ee*^*G*2619^ mutants, there are no changes in single evoked currents in Syn triple knock outs (Gitler et al., 2008).

Presynaptic exo- and endocytosis are thought to be coupled to maintain appropriate protein localization, preserve the structure of the synapse, and enable continued exocytosis (Maritzen and Haucke, 2018). Loss of function mutations in both *tsp42Ee* and *tsp42Eg* lead to reduced mEJC frequencies (Figure 2D) and evoked EJCs and quantal content in *tsp42Eg*^*MB*08050^ but not *tsp42Ee*^*G*2619^ mutants (Figures 2B,C). Both *tsp* mutants also exhibited increased endocytosis (Figure 4A) suggesting an uncoupling of exo- and endocytosis. Further, *tsp42Eg* mutants showed potentiated evoked responses during 20 Hz high frequency stimulation. Reduced evoked responses from a single stimulus but increased evoked responses during high frequency stimulation could occur because of altered Ca^2+^ and/or K^+^ dynamics.

Presynaptic exocytosis requires Ca^2+^ influx through voltage-gated Ca^2+^ channels thereby increasing intracellular Ca^2+^ at the AZ. Ca^2+^ binding to Syt enables the fusion and exocytosis of vesicles (Hackett and Ueda, 2015). Similarly, endocytosis also requires Ca^2+^ influx (Augustine et al., 2003), which occurs at AZs and periactive zones by Ca_v_2 and Ca_v_1 channels, respectively (Krick et al., 2021). The loss of Syt in both *tsp42Ee*^*G*2619^ and *tsp42Eg*^*MB*08050^ mutants (Figure 3C) may result in fewer functional release sites, despite an increase in Brp-positive puncta (Figure 3A), leading to reductions in mEJC frequency and evoked responses to a single suprathreshold stimulus in *tsp42Eg*^*MB*08050^ mutants. Because paired pulse ratios in both *tsp* mutants were similar as controls (Supplementary Figure 4), Ca^2+^ entry through properly localized AZ voltage-gated Ca^2+^ channels and Ca^2+^ sensitivity are probably unaffected in *tsp* mutants. Impaired Ca^2+^ buffering and/or extrusion, however, could potentially overcome the loss of Syt and result in increased Ca^2+^ accumulation during high frequency stimulation, enhanced evoked currents during high frequency stimulation, and enhanced endocytosis as observed in *tsp42Eg*^*MB*08050^ mutants (Figures 4A, 5A,B). Cbp53E is the sole Ca^2+^ buffer at *Drosophila* neuronal synapses including the NMJ (Hagel et al., 2015). In addition to Ca^2+^ buffers, synaptic Ca^2+^ is taken up by mitochondria and extruded by plasma membrane Ca^2+^ ATPases (PMCA). The latter is primarily responsible for Ca^2+^ clearance in *Drosophila* NMJ boutons after both single and trains of action potentials (Lnenicka et al., 2006). Thus, Cbp53E and/or PMCA may be deficient or mislocalized at *tsp* mutant synapses resulting in enhanced evoked responses during HFS and endocytosis.

Increased intracellular Ca^2+^ accumulation could also occur because of increased EndoA in *tsp* mutant NMJs (Figure 4B). Mammalian EndoAs are involved in multiple steps of endocytosis including the invagination of coated pits, the recruitment of Dyn to the neck, and the recruitment of Synaptojanin (Kjaerulff et al., 2011), which initiates uncoating of the vesicle. EndoA also interacts with Intersectin/Dap160 to facilitate vesicle priming and fusion in chromaffin neurosecretory cells (Gowrisankaran et al., 2020) and promotes Ca^2+^ channel clustering and Ca^2+^ influx in inner hair cell ribbon synapses (Kroll et al., 2019). In mouse hippocampal cells, overexpression of Endophilin A1 increases the release probability of vesicles (Weston et al., 2011).

Alternatively, reduced evoked responses from a single stimulus but increased evoked responses during high frequency stimulation could occur because of the loss of PI(4,5)P_2_ in *tsp42Eg*^*MB*08050^ mutants (Figure 6A). PI(4,5)P_2_ associates with synaptic proteins that include PDZ and pleckstrin homology (PH) domains and, through ionic interactions, receptors, ion channels, and cytoskeletal proteins (Katan and Cockcroft, 2020). PI(4,5)P_2_-rich regions of the membrane are bound by Syt1 (Park et al., 2015) and PI(4,5)P_2_ promotes, even in the absence of Ca^2+^, the membrane insertion of Syt1 (Bradberry et al., 2019). Notably, depletion of PI(4,5)P_2_ reduces inward K^+^ currents through K_v_7.2 channels in HEK cells (Gomis-Perez et al., 2017). K_v_7.2 and K_v_7.3 are subunits of voltage-gated K^+^ channel that progressively open during membrane depolarization to enable repolarization resulting in reduced excitability (Brown et al., 2007). Loss of function mutations in the genes encoding K_v_7.2 and K_v_7.3, *KCNQ2* and *KCNQ3*, respectively, are associated with hyperexcitability and seizure activity in animal models and humans (Nappi et al., 2020). Thus, the loss of PI(4,5)P_2_ in *tsp42Eg*^*MB*08050^ mutants may increase evoked responses during high frequency stimulation without affecting the size of vesicle pools due to a reduction in inward K^+^ currents.

The actin and microtubule cytoskeletons influence synaptic structure, neurotransmission, and endocytosis (Wu et al., 2016; Maritzen and Haucke, 2018; Piriya Ananda Babu et al., 2020). Actin polymers are enriched near both AZs and periactive zones (Kudryashova, 2021) and promote vesicle exocytosis (Guzman et al., 2019) and recycling (Dason et al., 2014). Our data suggest that actin polymerization is impaired at *tsp* mutant NMJs. Consistent with this, decreased PI(4,5)P_2_ levels, as we observed in *tsp42Eg*^*MB*08050^ mutants (Figure 6A), are correlated with decreased actin stability (Katan and Cockcroft, 2020). SCAR, which promotes actin nucleation and branching by activating Arp 2/3 (Zallen et al., 2002; Schenck et al., 2004), is reduced in both *tsp* mutants (Figure 6C). Actin associates with Syn (Bloom et al., 2003), which plays a role in maintaining (Zhang and Augustine, 2021) and releasing the RP vesicles to replenish the RRP during exocytosis (Akbergenova and Bykhovskaia, 2007; Shupliakov et al., 2011; Vasileva et al., 2012). Thus, altered actin dynamics in *tsp42Eg*^*MB*08050^ mutants may promote neurotransmitter release during high frequency stimulation. Inhibition of actin polymerization in cultured rat hippocampal neurons increases the amplitude of EPSCs (Morales et al., 2000). Similarly, the microtubule interacting protein Futsch/MAP1B is localized between microtubules and AZs where it is associated with Ca_v_1/Cacophony channels and Brp (Lepicard et al., 2014). Increased Futsch (Figure 6B) and Syn (Figure 3E) coupled with decreased actin stability at *tsp42Eg*^*MB*08050^ mutant NMJs may allow for more vesicles to be released upon intense stimulation.

**Figure 8:**
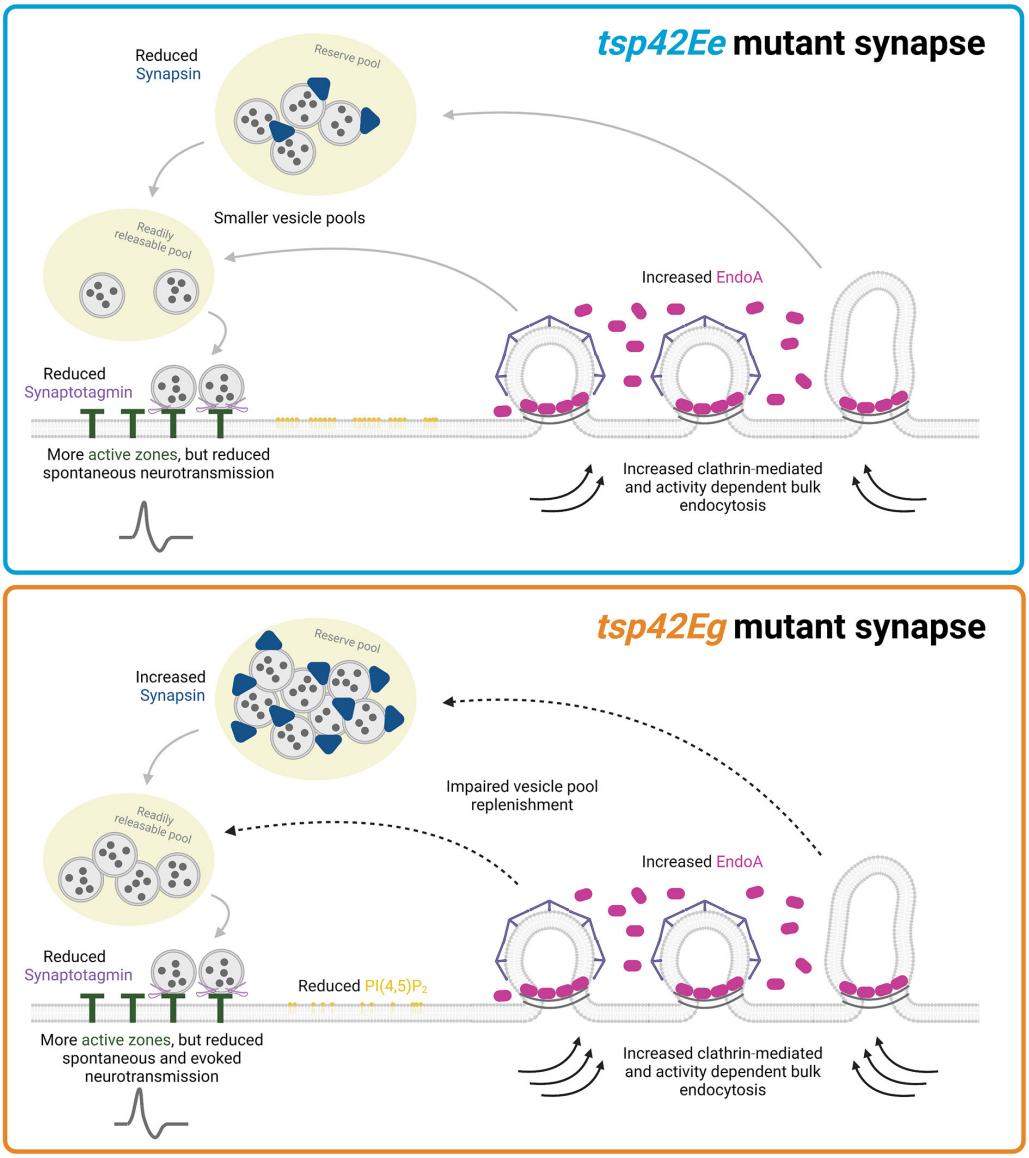
Tsp42Ee and Tsp42Eg regulate synaptic function through shared and distinct mechanisms. Both Tsp42Ee and Tsp42Eg negatively regulate endocytosis and synaptic localization of the endocytic protein, EndoA. However, Tsp42Ee and Tsp42Eg differentially affect Synapsin and vesicle pool dynamics. Tsp42Ee promotes synaptic localization of Synapsin and is implicated in the maintenance of vesicle pool size and mobilization of vesicles from the reserve pool to the readily releasable pool. Tsp42Eg, however, restricts Synapsin and is necessary for vesicle recycling. Tsp42Eg also distinctly regulates PI(4,5)P_2_ localization. Finally, both Tsp42Ee and Tsp42Eg facilitate active zone organization and promote Synaptotagmin localization to active zones. The combined synaptic functions of Tsp42Ee and Tsp42Eg allow for proper neurotransmission as *tsp42Ee*^*G*2619^ mutants have reduced spontaneous neurotransmission and *tsp42Eg*^*MB*08050^ mutants have reduced spontaneous and evoked release. Figure created with BioRender.com.

Collectively, our data suggest that Tsp42Eg restricts endocytosis, EndoA, Syn, and evoked release during HFS. Tsp42Eg may influence these synaptic characteristics by regulating synaptic PI(4,5)P_2_, and polymerization of the actin and microtubule cytoskeletons. Alternatively, Tsp42Ee restricts endocytosis and EndoA but promotes the synaptic localization of Syn thereby maintaining the total vesicle pool (Figure 8). Thus, our findings highlight both shared and distinct mechanisms through which Tsp42Ee and Tsp42Eg regulate synaptic function.

## Data availability statement

The raw data supporting the conclusions of this article will be made available by the authors, without undue reservation.

## Author contributions

EH and FL designed and performed experiments, analyzed data, prepared figures, and wrote and edited the manuscript. IS, BP, and FB performed experiments, analyzed data, and collaborated on writing the methods. All authors contributed to this manuscript and approved of the submitted version.

## Funding

This work was supported by the National Institution of Health Grant, NINDS 1R15NS101608-01A1, to FL and the Southern Illinois University Graduate School Competitive Graduate Award to EH.

## Conflict of interest

The authors declare that the research was conducted in the absence of any commercial or financial relationships that could be construed as a potential conflict of interest.

## Publisher's note

All claims expressed in this article are solely those of the authors and do not necessarily represent those of their affiliated organizations, or those of the publisher, the editors and the reviewers. Any product that may be evaluated in this article, or claim that may be made by its manufacturer, is not guaranteed or endorsed by the publisher.

## Supplementary material

Supplementary Figure 1 Survival curves of *tsp* mutants are significantly different from control (*w*^1118^) animals (*tsp42Ee*^*G*2619^, *p* = 0.0003 and *tsp42Eg*^*MB*08050^, *p* = 0.0222). Log-rank (Mantel-Cox) tests were used for survival curve comparison. Shaded regions indicate 95% confidence intervals.

Supplementary Figure 2 Expression of the reference transcripts, *brp, dyn*, and *rab11*, do not differ in *tsp42Ee*^*G*2619^ or *tsp42Eg*^*MB*08050^ mutants. Reference transcripts were assessed in CNS, muscle (*dyn* and *rab11* only), and all tissues of *tsp* mutants. There were no differences in expression of *brp, dyn*, and *rab11* in *tsp* mutants in any tissue type. Expression is shown in all tissues relative to controls (*w*^1118^).

Supplementary Figure 3 Synaptic levels of vGLUT are similar in *tsp* mutants and controls. High resolution confocal images of *w*^1118^ (control), *tsp42Ee*^*G*2619^ mutant, or *tsp42Eg*^*MB*08050^ mutant NMJs. Synaptic vGLUT (green, bottom left panels) does not differ between controls and *tsp* mutants (right bar graph). Scale bar = 5 μM.

Supplementary Figure 4 Paired pulse ratios are similar in *tsp42Ee*^*G*2619^ and *tsp42Eg*^*MB*08050^ mutants compared with controls (*w*^1118^). Paired pulse ratios were obtained in a bath solution containing 1.0 mM Ca^2+^ and calculated by dividing the amplitude of the first evoked response by the amplitude of the second evoked response.

Supplementary Figure 5 FM 1-43FX does not preferentially adhere to *tsp* mutant membranes. Genotypes were dissected in HL-3 without Ca^2+^. Subsequently animals were either stimulated with 90 mM KCl for 1 min (bottom panels) or the HL-3 was replaced (top panels) in the presence of 4 μM FM 1-43FX and 1.0 mM Ca^2+^. Scale bar = 5 μM.
