# Supplementary figures and images for: The CD63 homologs, Tsp42Ee and Tsp42Eg, restrict endocytosis and promote neurotransmission through differential regulation of synaptic vesicle pools
Source: Front Cell Neurosci. 2022 Aug 22;16:957232. doi: 10.3389/fncel.2022.957232 (PMC9441712; doi:10.3389/fncel.2022.957232)

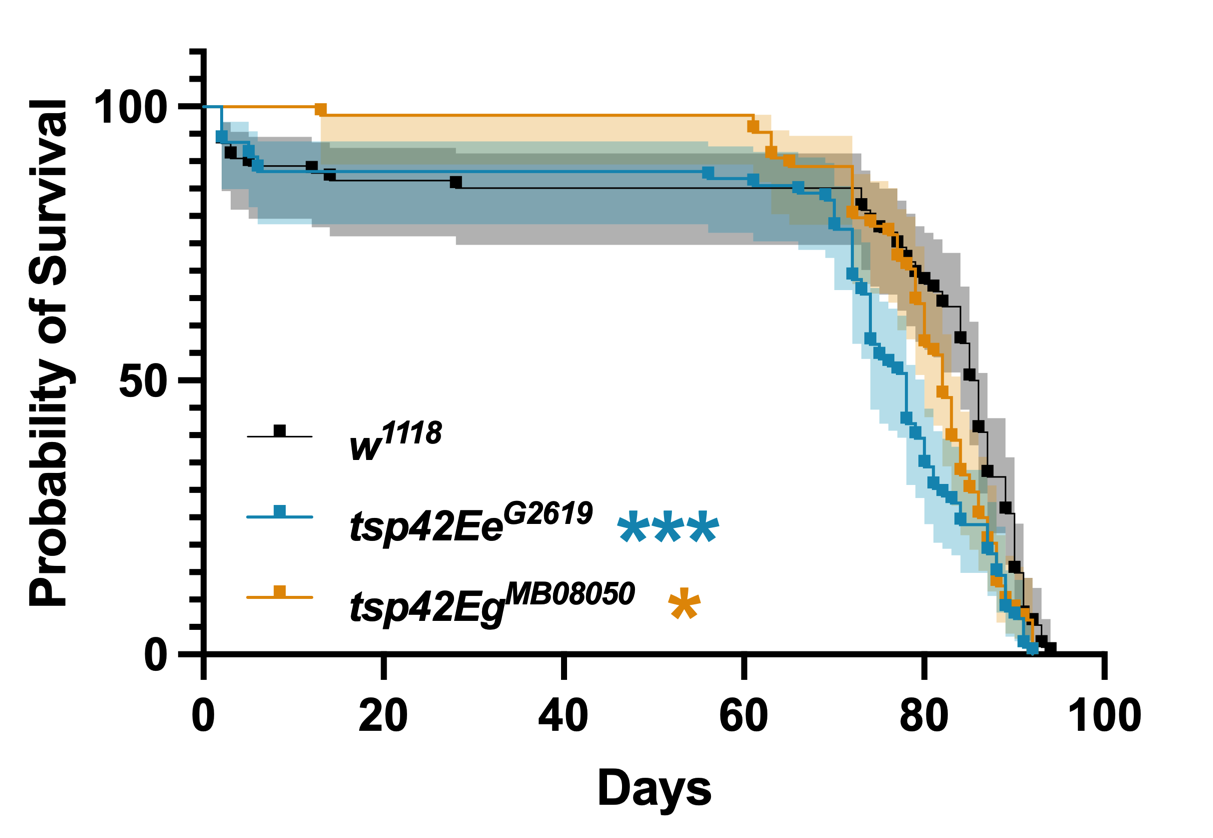

Supplement: Supplementary Figure 1 — Survival curves of tsp mutants are significantly different from control (w1118) animals (tsp42EeG2619, p = 0.0003 and tsp42EgMB08050, p = 0.0222). Log-rank (Mantel-Cox) tests were used for survival curve comparison. Shaded regions indicate 95% confidence intervals. [file Image_1.TIFF]

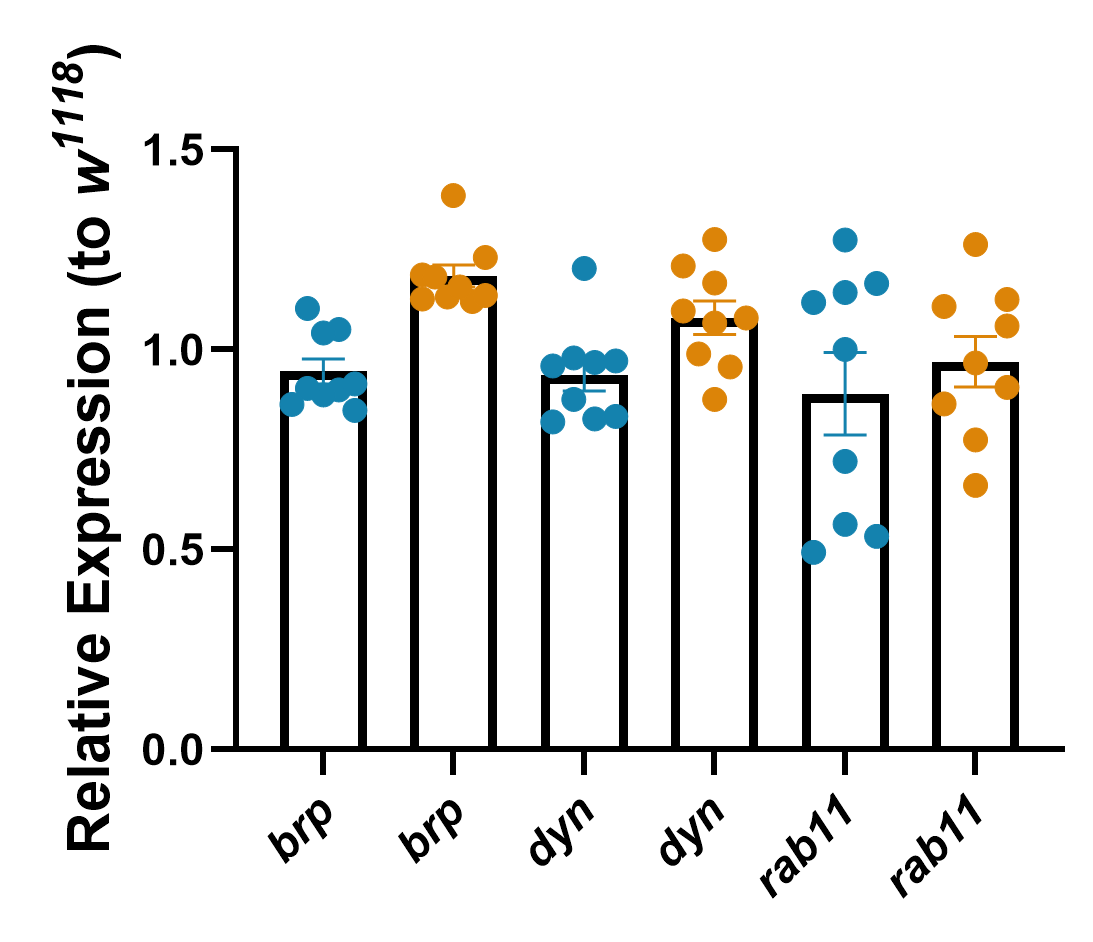

Supplement: Supplementary Figure 2 — Expression of the reference transcripts, brp, dyn, and rab11, do not differ in tsp42EeG2619 or tsp42EgMB08050 mutants. Reference transcripts were assessed in CNS, muscle (dyn and rab11 only), and all tissues of tsp mutants. There were no differences in expression of brp, dyn, and rab11 in tsp mutants in any tissue type. Expression is shown in all tissues relative to controls (w1118). [file Image_2.TIF]

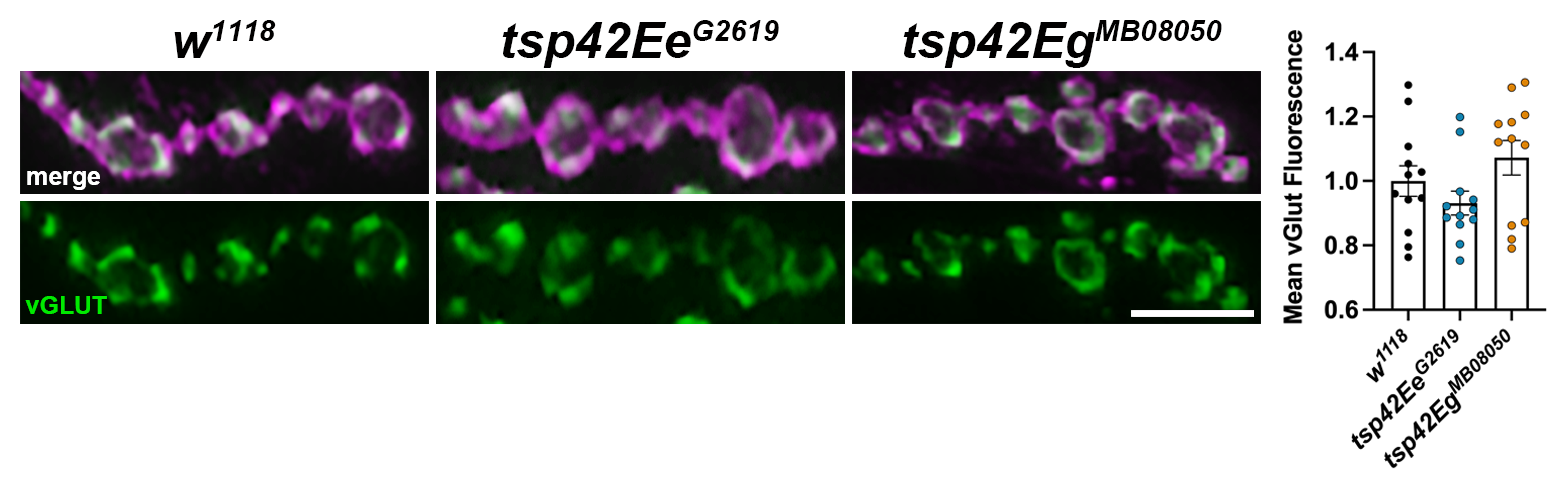

Supplement: Supplementary Figure 3 — Synaptic levels of vGLUT are similar in tsp mutants and controls. High resolution confocal images of w1118 (control), tsp42EeG2619 mutant, or tsp42EgMB08050 mutant NMJs. Synaptic vGLUT (green, bottom left panels) does not differ between controls and tsp mutants (right bar graph). Scale bar = 5 μM. [file Image_3.TIF]

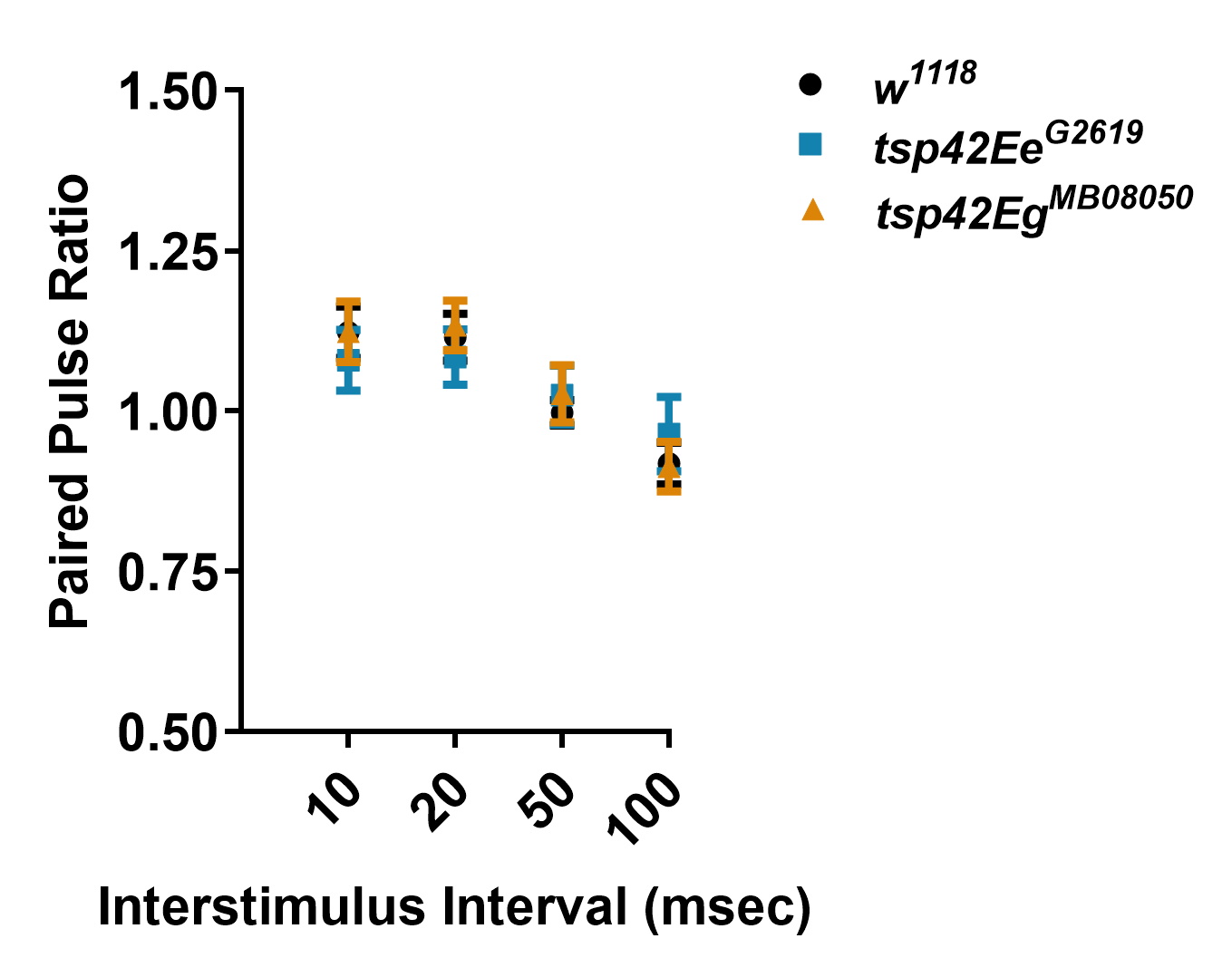

Supplement: Supplementary Figure 4 — Paired pulse ratios are similar in tsp42EeG2619 and tsp42EgMB08050 mutants compared with controls (w1118). Paired pulse ratios were obtained in a bath solution containing 1.0 mM Ca2+ and calculated by dividing the amplitude of the first evoked response by the amplitude of the second evoked response. [file Image_4.TIF]

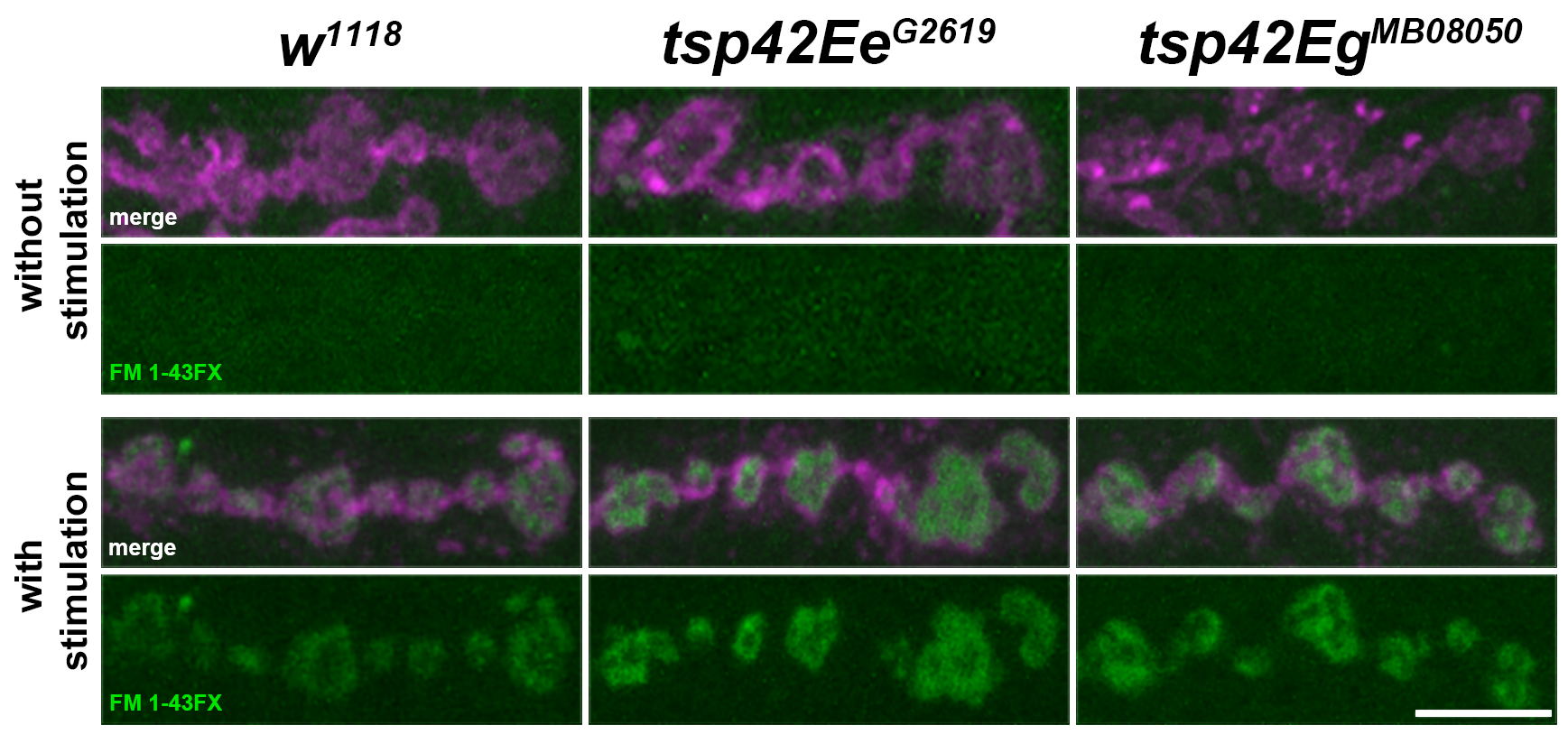

Supplement: Supplementary Figure 5 — FM 1-43FX does not preferentially adhere to tsp mutant membranes. Genotypes were dissected in HL-3 without Ca2+. Subsequently animals were either stimulated with 90 mM KCl for 1 min (bottom panels) or the HL-3 was replaced (top panels) in the presence of 4 μM FM 1-43FX and 1.0 mM Ca2+. Scale bar = 5 μM. [file Image_5.TIF]
